# Preparation of optically active bicyclodihydrosiloles by a radical cascade reaction

**DOI:** 10.3762/bjoc.9.149

**Published:** 2013-07-04

**Authors:** Koichiro Miyazaki, Yu Yamane, Ryuichiro Yo, Hidemitsu Uno, Akio Kamimura

**Affiliations:** 1 Department of Applied Molecular Bioscience, Graduate School of Medicine, Yamaguchi University, Ube 755-8611, Japan; 2 Department of Chemistry, Graduate School of Science and Engineering, Ehime University, Matsuyama, 790-8577, Japan

**Keywords:** bicyclodihydrosilole, free radical, radical cascade reaction, S_H_i reaction, tris(trimethylsilyl)silane

## Abstract

Bicyclodihydrosiloles were readily prepared from optically active enyne compounds by a radical cascade reaction triggered by tris(trimethylsilyl)silane ((Me_3_Si)_3_SiH). The reaction was initiated by the addition of a silyl radical to an α,β-unsaturated ester, forming an α-carbonyl radical that underwent radical cyclization to a terminal alkyne unit. The resulting vinyl radical attacked the silicon atom in an S_H_i manner to give dihydrosilole. The reaction preferentially formed *trans* isomers of bicyclosiloles with an approximately 7:3 to 9:1 selectivity.

## Introduction

Radical cyclization occupies a unique position in organic synthesis because it is a useful reaction for the construction of cyclic molecules [1–10]. The radical cascade cyclization process is also an interesting synthetic reaction that often provides an efficient method [11–13]. Recently, we reported a new type of higher-order radical cascade reaction between chiral enyne compounds and Bu_3_SnH, which is recognized as a useful reagent in radical reactions [14]. In this reaction, radical addition–cyclization cascade followed by intramolecular radical substitution (S_H_i) occurred in one-pot to give optically active bicyclostannolanes in good yields [15]. We are interested in whether such a cascade S_H_i process might occur with other radical species. We have found that a methylthiyl radical also undergoes such a radical cascade reaction to stereoselectively give bicyclic dihydrothiophenes [16]. We expected that tris(trimethylsilyl)silane (Me_3_Si)_3_SiH [17], which is a well-known alternative to Bu_3_SnH in radical reactions [18–22], would be a good promoter of a similar cascade S_H_i reaction, because there were several reports so far that show such S_H_i reaction on silicon atoms progressing efficiently [23–28]. In this paper, we report a new synthesis of chiral bicyclodihydrosiloles through an addition–cyclization–S_H_i cascade reaction in one-pot treatment of chiral enyne compounds. A good *trans*-selectivity was observed in the reaction.

## Results and Discussion

We examined the cascade process using optically active enyne precursor **1a**, which was prepared by a Michael/aldol domino reaction to chiral sulfinimines followed by thermal elimination and *N*-propargylation [29–30]. We first optimized the reaction conditions. The results are summarized in Table 1.

**Table 1:** Radical cascade reaction under various reaction conditions.

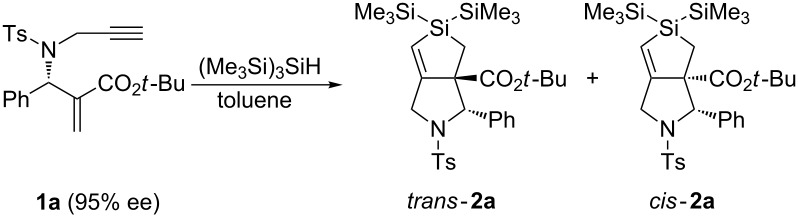				

Entry	Initiator (equiv)	Temp (°C)	**2a**; Yield (%)^a^	*trans*/*cis*^b^

1	AIBN (0.1)	110	14	n/a
2	AIBN (1.0)	110	39	69/31
3	Et_3_B (3.0)	25	58	80/20 (95)^c^
4	Et_3_B (3.0)	0	48	86/14

^a^Isolated yield. ^b^Determined by HPLC analyses. ^c^Enantiomeric excess for *trans*-**2a**. Determined by chiral HPLC analysis using ChiralPak ID.

Treatment of **1** with (Me_3_Si)_3_SiH in the presence of catalytic amounts of AIBN at 110 °C resulted in the formation of the desired bicyclodihydrosilole **2a** in 14% yield (Table 1, entry 1). The use of one equivalent of AIBN improved the yield of **2a** to 39% (Table 1, entry 2). These results suggest that the radical chain reaction insufficiently progressed during the reaction initiated by AIBN. The product contained two diastereomers, which were separated by chromatography. The use of Et_3_B/air as an initiator enhanced the yield of **2a** to 58% (Table 1, entry 3). The enantiomeric excess of *trans***-2a** was estimated to be 95% by HPLC analysis, which was the same ee level of precursor **1a**. Thus, no epimerization at the C3 chiral center occurred during the reaction. The stereoselectivity was improved to 8:2. The stereoselectivity was sensitive to the reaction temperature, and an 86/14 mixture of *trans****-*2a** and *cis***-2a** was obtained when the reaction was performed at 0 °C, although the yield was less than that obtained when the reaction was performed at room temperature (Table 1, entry 4).

Having determined the optimized reaction conditions, we examined the generality of the reaction. The results are summarized in Table 2.

**Table 2:** Preparation of pyrrolidinodihydrosiloles **2**.

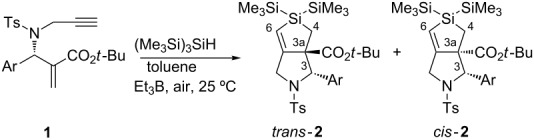					

Entry	Ar	Product	Yield^a^ (%)	*trans*/*cis*^b^	ee for *trans*-**2**^c^

1	2-MeC_6_H_4_	**2b**	60	84/16	nd^d^
2	4-MeC_6_H_4_	**2c**	53	91/9	nd^d^
3	4-MeOC_6_H_4_	**2d**	42	86/14	97
4	3-ClC_6_H_4_	**2e**	42	71/29	nd^d^
5	4-ClC_6_H_4_	**2f**	51	81/19	90
6	4-FC_6_H_4_	**2g**	61	80/20	97
7	4-CF_3_C_6_H_4_	**2h**	61	80/20	68
8	2-thienyl	**2i**	48	75/25	98
9	2-naphthyl	**2j**	51	81/19	99

^a^Isolated yield. ^b^Determined by HPLC analyses. ^c^Determined by HPLC analyses with a Chiral-Pak-ID. ^d^Not determined owing to insufficient separation by chiral HPLC analyses with ChiralPak ID and IC.

For example, the reaction of **1b** smoothly occurred, giving bicyclic dihydrosilole **2b** in 60% yield. HPLC analysis of the reaction mixture revealed that the diastereomeric ratio of **2b** was 84/16. Dihydrosiloles **2c**–**2j** were isolated in good yields from other precursors in a *trans*-selective manner (Table 2, entries 2–9). Their diastereomeric ratios ranged from 9/1 to 7/3. Although we could not determine the enantiomeric excesses for some compounds of **2** because of insufficient separation by chiral HPLC analyses using ChiralPak ID and IC (Table 2, entries 1, 2, and 4), the enantiomeric excesses of most of products **2** were high, and their original values were maintained (Table 2, entries 3, 5, 6, 8, and 9). Interestingly, significant epimerization occurred during the reaction of **1h**; the enantiomeric excess of **2h** was only 68% ee (Table 2, entry 7).

**Figure 1:**
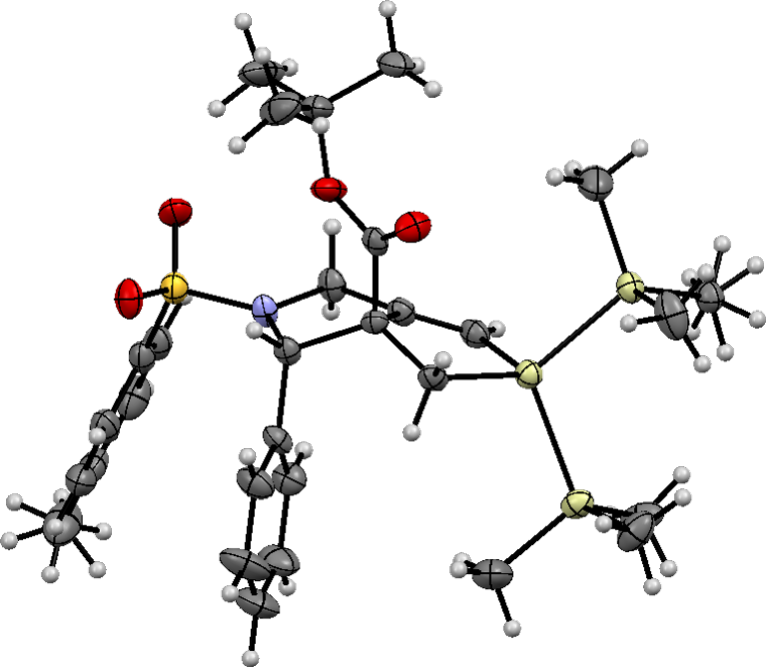
ORTEP structure of *trans*-**2a**.

The configuration of **2** was determined in the following manner: The major isomer of **2a** was highly crystalline, which allowed the performance of X-ray crystallography. The observed data clearly showed a *trans-* **2a** structure [31]. The ORTEP structure of major **2a**, which unambiguously indicates a *trans* configuration, is shown in Figure 1. The ^1^H NMR spectra of *trans-***2a** and other major **2** showed similar trends, and *trans* configurations for other major **2** were determined unambiguously.

Unfortunately, none of the minor **2** formed suitable crystals, which precluded X-ray analysis of the minor isomers. However, their ^1^H NMR spectra showed several diagnostic points. For example, the *tert*-butyl group in the ester at the C3a position in minor **2a** appeared at 1.17 ppm; this peak was substantially shifted toward higher field than *trans*-**2a**. Compared with X-ray data for the sulfur analogue of *cis*-**2a**, the *tert*-butyl ester group is located above the aromatic ring at C3, and expected to introduce an anisotropic effect that subsequently causes a high-field shift for the *tert*-butyl protons [28]. Other typical differences between the ^1^H NMR spectra of minor **2a** and major **2a** (= *trans*-**2a**) included the following: The peaks of the CH_2_Si group at the C4 position in minor **2a** appeared at 0.92 and 2.00 ppm, whereas the corresponding peaks of *trans***-2a** were observed at 0.49 and 1.14 ppm. In addition, we found that H6 and H3 appeared at 5.51 and 4.46 ppm, respectively, in the spectrum of minor **2a**. The corresponding protons in *trans***-2a** appeared at substantially lower-field positions at 5.86 and 5.53 ppm. We assumed that this shift was caused by another anisotropic effect of the Ts group at N2. These trends in the ^1^H NMR spectra were also observed in the sulfur analogues of *cis*-**2**. Thus, we concluded that the minor isomer of **2** exhibited *cis* configuration.

**Scheme 1:**
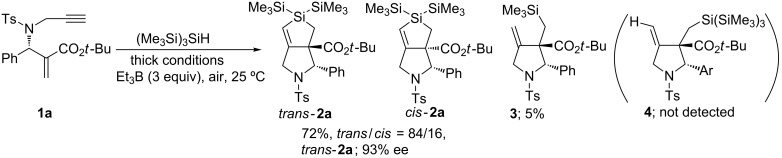
Formation of bicyclic dihydrosilole **2a** under high concentration conditions.

To explore the reaction mechanism, we examined the reaction of **1a** without additional solvents (Scheme 1).

The treatment of **1a**, (Me_3_Si)_3_SiH, and Et_3_B in hexane under an air atmosphere gave **2a** in 72%. To our surprise, this yield was better than that of the reaction performed under the usual conditions. We expected that *exo*-methylenepyrrolidine **4** would be a side product under these conditions, and we indeed detected an *exo*-methylene compound in 5% yield in the reaction mixture. However, NMR spectra and HRMS results indicated that the isolated product was compound **3,** which contained a Me_3_SiCH_2_- group instead of a (Me_3_Si)_3_SiCH_2_- group. These results suggest that Me_3_Si radicals were generated during the cascade reaction, and that a small part of the radical was subsequently trapped by **1** under such conditions.

**Scheme 2:**
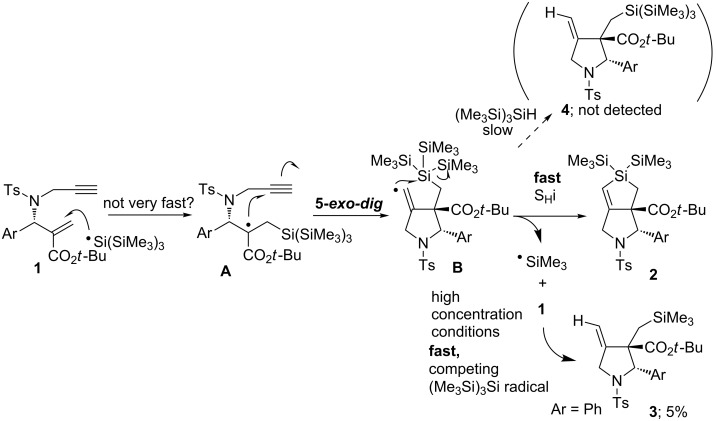
Plausible reaction mechanism.

We believe this process progressed in a similar manner to our previously investigated reaction that involved tributyltin radicals [15]. A plausible reaction mechanism is depicted in Scheme 2.

The (Me_3_Si)_3_Si radical attacks the β-carbon of the α,β-unsaturated ester in **1,** and α-carbonyl radical **A** is generated. Intermediate **A** undergoes radical cyclization in a 5-*exo*-*dig* mode giving vinyl radical intermediate **B**, which is potentially reactive for attacking the silyl group in an S_H_i manner to give a Me_3_Si radical and **2**. The process from **B** to **2** should be very rapid. Giese and co-workers have reported that the reaction rate for a similar S_H_i process reaches 2.4 × 10^5^ s^−1^ at 80 °C [25]. Although most of the Me_3_Si radicals undergo hydrogen abstraction from (Me_3_Si)_3_SiH to yield a new (Me_3_Si)_3_Si radical and Me_3_SiH, a small fraction of the Me_3_Si radicals compete to attack **1**; a similar cascade reaction progresses consequently, and compound **3** is formed in 5% yield under very high concentration conditions. We assume that compound **4** was not detected in the reaction product under such conditions for two reasons: first, as previously mentioned, the S_H_i process from intermediate **B** to **2** is very rapid, and the process occurs faster than intermolecular hydrogen abstraction from (Me_3_Si)_3_SiH, even under high concentration conditions. Second, the addition rate of (Me_3_Si)_3_Si radicals to alkenes should be relatively slow; the rate competes with the addition rate of Me_3_Si radicals to alkenes. This reason is supported by the results that indicated the yield of **2a** to be much improved under high (Me_3_Si)_3_SiH concentration conditions because the addition rate should be accelerated as the concentration of (Me_3_Si)_3_SiH increased.

**Scheme 3:**
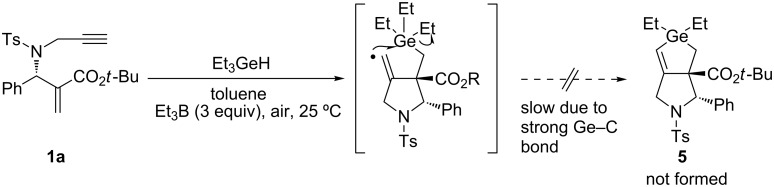
Reaction **1a** with Et_3_GeH.

We examined whether a germyl radical might undergo a similar reaction with **1**. Treatment of **1** with Et_3_GeH in the presence of Et_3_B, however, failed in the formation of the corresponding compound **5**. This failure was probably because a carbon–germanium bond, which is supposed to be stronger than a Si–Si bond, was never cleaved under these conditions (Scheme 3). Another possibility of this failure might be that the addition rate of a triethylgermyl radical to enyne **1a** was slow and less efficient.

## Conclusion

In conclusion, we have successfully converted chiral enyne compounds **1**, which were readily available from an asymmetric aza-Morita–Baylis–Hillman equivalent reaction, into bicyclic pyrolidinodihydrosiloles **2** in good yields. These reactions progressed in a highly stereoselective manner. Further application of the present silole synthesis is now underway in our laboratory.

## Experimental

**General methods:** All ^1^H and ^13^C NMR spectra were recorded on a JEOL JNM-ECA500 Delta2 (500 MHz for ^1^H, 126 MHz for ^13^C) spectrometer. All the reactions in this study were performed under nitrogen atmosphere unless otherwise noted. CH_2_Cl_2_ was dried over CaH_2_, and distilled under nitrogen before use. High-resolution mass spectra (HRMS) were measured at the Tokiwa Instrumentation Analysis Center, Yamaguchi University.

**Preparation of (3*S*)-*tert*-butyl 3-phenyl-2-tosyl-5,5-bis(trimethylsilyl)-1,2,3,3a,4,5-hexahydrosilolo[3,4-*c*]pyrrole-3a-carboxylate (2a)**. A solution of **1a** (85 mg, 0.201 mmol, 95% ee), (Me_3_Si)_3_SiH (0.06 mL, 0.195 mmol), and Et_3_B (1.0 M in hexane, 0.60 mL, 0.60 mmol) in toluene (20 mL) was stirred at room temperature under air for 15 min. The reaction mixture was concentrated in vacuo, and the residue was purified by flash chromatography (silica gel/hexane–EtOAc 15/1 to 10/1, v/v) to give **2a** in 58% yield (70.2 mg, 0.117 mmol). The two diastereomers, *trans***-2a** and *cis****-*2a**, were separated by further careful chromatography.

**(3*S*,3a*S*)-*tert*-Butyl 3-phenyl-2-tosyl-5,5-bis(trimethylsilyl)-1,2,3,3a,4,5-hexahydrosilolo[3,4-*c*]pyrrole-3a-carboxylate (*trans*-2a).** White solid; mp 144–145 °C; [α]_D_ −31.8 (*c* 0.68, CHCl_3_); the enantiomeric purity was determined by HPLC analysis, *t*_R_ 10.0 min (major), *t*_R_ 11.5 min (minor) [CHIRALPAK ID (0.46 cm × 25 cm), hexane/iPrOH, 95/5, 40 °C, 1.0 mL/min] to be 95% ee; ^1^H NMR (500 MHz, CHCl_3_) δ 7.32 (d, *J* = 8.2 Hz, 2H), 7.26 (s, 3H), 7.24–7.07 (m, 2H), 7.03 (d, *J* = 7.8 Hz, 2H), 5.86 (s, 1H), 5.23 (s, 1H), 4.42 (d, *J* = 13.0 Hz, 1H), 3.95 (d, *J* = 13.0 Hz, 1H), 2.32 (s, 3H), 1.51 (s, 9H), 1.15 (d, *J* = 14.9 Hz, 1H), 0.50 (d, *J* = 14.8 Hz, 1H), 0.07 (s, 9H), −0.20 (s, 9H); ^13^C NMR (126 MHz, CHCl_3_) δ 173.9, 157.6, 142.6, 138.6, 137.1, 129.1 (2C), 128.3 (br, 4C), 127.5, 127.0 (2C), 124.2, 82.3, 71.1, 69.7, 50.5, 28.0 (3C), 21.5, 12.2, −0.3 (3C), −0.9 (3C); HRMS–ESI (positive mode; M + Na) *m*/*z* 622.2282, calcd for C_30_H_45_NNaO_4_SSi_3_, 622.2275.

**(3*S*,3a*R*)-*tert*-Butyl 3-phenyl-2-tosyl-5,5-bis(trimethylsilyl)-1,2,3,3a,4,5-hexahydrosilolo[3,4-c]pyrrole-3a-carboxylate (*cis*-2a).** Pale yellow oil; [α]_D_ +97.3 (c 0.27, CHCl_3_); ^1^H NMR (500 MHz, CHCl_3_) δ 7.63 (d, *J* = 7.8 Hz, 2H), 7.57–7.50 (m, 2H), 7.33–7.22 (m, 5H), 5.51 (s, 1H), 4.60 (d, *J* = 14.3 Hz, 1H), 4.23 (s, 1H), 4.11 (dd, *J* = 14.3, 1.6 Hz, 1H), 2.39 (s, 3H), 2.00 (d, *J* = 12.8 Hz, 1H), 1.17 (s, 9H), 0.92 (d, *J* = 15.0 Hz, 1H), 0.04 (s, 9H), −0.11 (s, 9H); ^13^C NMR (126 MHz, CHCl_3_) δ 169.5, 157.8, 143.8, 138.1, 133.1, 129.9 (2C), 128.0 (2C), 127.7 (2C), 127.7, 127.1 (br, 2C), 122.8, 82.1, 75.2, 72.5, 53.7, 27.9 (3C), 21.6, 17.4, 0.3 (3C), −1.4 (3C); HRMS–ESI (positive mode; M + Na) *m*/*z* 622.2292, calcd for C_30_H_45_NNaO_4_SSi_3_, 622.2275.

**Preparation of 2a under no solvent conditions** (Scheme 3, neat condition). A solution of **1a** (85 mg, 0.201 mmol), (Me_3_Si)_3_SiH (0.07 mL, 0.228 mmol), and Et_3_B (1.0 M in hexane, 0.60 mL, 0.60 mmol) was stirred at room temperature for 15 min under air. The reaction mixture was concentrated in vacuo, and the yellow residue was purified by flash chromatography (silica gel/hexane–EtOAc 30/1 to 20/1 v/v) to give **2a** in 72% yield (85.6 mg, 0.143 mmol). The *trans****-*2a**/*cis****-*2a** ratio was determined to be 84/16. Careful separation of these two diastereomers gave pure *trans***-2a** and minor isomers that contained *cis***-2a** and **3** in a 74/26 ratio. The separation of **3** was achieved using a recycling GPC apparatus, giving pure **3** in 5% yield (5.1 mg, 0.011 mmol).

**(2*S*,3*S*)-*tert*-Butyl 3-((trimethylsilyl)methyl)-4-methylene-2-phenyl-1-tosylpyrrolidine-3-carboxylate (3).** Pale yellow oil; [α]_D_ +3.0 (c 0.01, CHCl_3_); ^1^H NMR (500 MHz, CHCl_3_) δ 7.20–7.09 (m, 5H), 7.00–6.94 (m, 4H), 5.36 (s, 1H), 5.21 (t, *J* = 1.8 Hz, 1H), 5.14 (dd, *J* = 2.7, 1.5 Hz, 1H), 4.36 (dt, *J* = 13.0, 2.5 Hz, 1H), 3.90 (dt, *J* = 13.0, 1.5 Hz, 1H), 2.29 (s, 3H), 1.50 (s, 9H), 0.90 (d, *J* = 14.6 Hz, 1H), 0.48 (d, *J* = 14.7 Hz, 1H), −0.13 (s, 9H); ^13^C NMR (126 MHz, CHCl_3_) δ 172.4, 148.8, 142.4, 138.2, 136.7, 129.0 (4C), 128.1 (2C), 127.8, 127.0 (2C), 110.0, 82.4, 70.2, 61.0, 51.5, 27.9 (3C), 21.5, 19.6, 0.7 (3C); HRMS–ESI (positive mode; M + Na) *m/z* 522.2108, calcd for C_27_H_37_NNaO_4_SSi, 522.2110.

## Supporting Information

File 1 Experimental procedures and ^1^H and ^13^C NMR spectra.

File 2 CIF data for *trans***-2a**.
