# Supplementary material for: Preparation of optically active bicyclodihydrosiloles by a radical cascade reaction
Source: Beilstein J Org Chem. 2013 Jul 4;9:1326–32. doi: 10.3762/bjoc.9.149 (PMC3740799; doi:10.3762/bjoc.9.149)

Supporting information

for

Preparation of optically active bicyclodihydrosiloles by a radical cascade reaction

Koichiro Miyazaki[1], Yu Yamane[1], Ryuichiro Yo[1], Hidemitsu Uno[2] and Akio Kamimura*,[1]

Address: [1]Department of Applied Molecular Bioscience, Graduate School of Medicine, Yamaguchi University, Ube 755-8611, Japan and [2]Department of Chemistry, Graduate School of Science and Engineering, Ehime University, Matsuyama, 790-8577, Japan

Email: Akio Kamimura - ak10@yamaguchi-u.ac.jp

*Corresponding author

Experimental procedures and ^{1}H and ^{13}C NMR spectra

Contents

Experimental S2

^{1}H and ^{13}C NMR spectra S8

Experimental

All ^{1}H and ^{13}C NMR spectra were recorded on a JEOL JNM-ECA500 Delta2 (500 MHz for ^{1}H, 126 MHz for ^{13}C) spectrometer. All the reactions in this study were performed under nitrogen atmosphere unless otherwise noted. CH_2Cl_2 was dried over CaH_2, and distilled under nitrogen before use. High-resolution mass spectra (HRMS) were measured at the Tokiwa Instrumentation Analysis Center, Yamaguchi University.

(3*S*,3a*S*)-*tert*-Butyl 3-(*o*-tolyl)-2-tosyl-5,5-bis(trimethylsilyl)-1,2,3,3a,4,5-hexahydrosilolo[3,4-*c*]pyrrole-3a-carboxylate (*trans* 2b). The reaction was performed in a similar manner to the preparation of **2a**, starting with **1b** (218.2 mg, 0.496 mmol) with $(Me_3Si)_3SiH$ (0.18 mL, 0.586 mmol) and Et_3B (1.0 M in hexane, 1.50 mL, 1.50 mmol) giving **2b** in 60% yield (183.3 mg, 0.299 mmol). Further chromatographic purification gave *trans* **2b**. Brown solid; mp: 49–50 °C; $[\alpha]_D$ –53.3 (*c* 1.04, $CHCl_3$); ^{1}H NMR (500 MHz, $CDCl_3$) δ 7.38 (d, *J* = 8.2 Hz, 2H), 7.12–7.00 (m, 5H), 6.79 (d, *J* = 9.2 Hz, 1H), 6.40 (d, *J* = 8.2 Hz, 1H), 5.87 (s, 1H), 5.55 (s, 1H), 4.41 (dd, *J* = 12.9, 2.2 Hz, 1H), 3.97 (dd, *J* = 12.7, 1.3 Hz, 1H), 2.38 (s, 3H), 2.32 (s, 3H), 1.49 (s, 9H), 1.17 (d, *J* = 14.8 Hz, 1H), 0.46 (d, *J* = 14.8 Hz, 1H), 0.06 (s, 9H), −0.24 (s, 9H); ^{13}C NMR (126 MHz, $CDCl_3$) δ 174.1, 157.4, 142.8, 137.1, 137.0, 136.3, 129.9, 129.2 (2C), 127.3, 127.1 (2C), 126.7, 126.3, 124.6, 82.2, 70.8, 65.6, 50.5, 28.0 (3C), 21.5, 19.9, 11.7, –0.3 (3C), –1.0 (3C); HRMS–ESI (positive mode; M + Na) *m*/*z* 636.2409, calcd for $C_{31}H_{47}NNaO_4SSi_3$, 636.2431.

(3*S*,3a*S*)-*tert*-Butyl 3-(*p*-tolyl)-2-tosyl-5,5-bis(trimethylsilyl)-1,2,3,3a,4,5-hexahydrosilolo[3,4-*c*]pyrrole-3a-carboxylate (*trans* 2c), The reaction was performed in a similar manner to the preparation of **2a**, starting with **1c** (83.7 mg, 0.191 mmol) with $(Me_3Si)_3SiH$ (0.07 mL, 0.228 mmol) and Et_3B (1.0 M in hexane,

0.60 mL, 0.60 mmol) giving **2c** in 53% yield (62.3 mg, 0.102 mmol). Further chromatographic purification gave *trans* **2c**. Colorless oil; $[\alpha]_D$ –81.6 (*c* 0.49, $CHCl_3$); ^1H NMR (500 MHz, $CDCl_3$) δ 7.34 (d, *J* = 8.3 Hz, 2H), 7.03 (d, *J* = 8.6 Hz, 2H), 7.00–6.61 (m, 4H), 5.85 (s, 1H), 5.18 (s, 1H), 4.38 (d, *J* = 12.9 Hz, 1H), 3.94 (d, *J* = 12.9 Hz, 1H), 2.32 (s, 3H), 2.26 (s, 3H), 1.49 (s, 9H), 1.12 (d, *J* = 15.0 Hz, 1H), 0.53 (d, *J* = 15.0 Hz, 1H), 0.06 (s, 9H), −0.20 (s, 9H); ^{13}C NMR (126 MHz, $CDCl_3$) δ 174.0, 157.7, 142.6, 137.2, 137.1, 135.6, 129.1 (2C), 129.0 (br, 4C), 127.1 (2C), 124.1, 82.1, 71.1, 69.5, 50.5, 28.0 (3C), 21.5, 21.1, 12.1, –0.3 (3C), –1.0 (3C); HRMS–ESI (positive mode; M + Na) *m*/*z* 636.2431, calcd for $C_{31}H_{47}NNaO_4SSi_3$, 636.2431.

(3*S*,3a*S*)-*tert*-Butyl 3-(4-methoxyphenyl)-2-tosyl-5,5-bis(trimethylsilyl)-1,2,3,3a,4,5-hexahydrosilolo[3,4-*c*]pyrrole-3a-carboxylate (*trans* 2d): The reaction was performed in a similar manner to the preparation of **2a**, starting with **1d** (90.9 mg, 0.200 mmol) with $(Me_3Si)_3SiH$ (0.07 mL, 0.228 mmol) and Et_3B (1.0 M in hexane, 0.80 mL, 0.80 mmol) giving **2d** in 42% yield (52.7 mg, 0.0838 mmol). Further chromatographic purification gave *trans*-**2d**. White solid; mp: 145–146 °C; $[\alpha]_D$ −84.3 (*c* 0.77, $CHCl_3$); the enantiomeric purity was determined by HPLC analysis, t_R 12.9 min (major), t_R 15.9 min (minor) [CHIRALPAK ID (0.46 cm × 25 cm) (from Daicel Chemical Ind., Ltd.) hexane/iPrOH, 95/5, 40 °C, 1.0 mL/ min] as 97% ee; ^1H NMR (500 MHz, $CDCl_3$) δ 7.32 (d, *J* = 8.2 Hz, 2H), 7.04 (d, *J* = 7.8 Hz, 2H), 6.83–6.42 (m, 4H), 5.84 (s, 1H), 5.17 (s, 1H), 4.39 (dd, *J* = 12.9, 2.2 Hz, 1H), 3.91 (dd, *J* = 13.8, 0.8 Hz, 1H), 3.74 (s, 3H), 2.32 (s, 3H), 1.49 (s, 9H), 1.13 (d, *J* = 14.9 Hz, 1H), 0.54 (d, *J* = 15.0 Hz, 1H), 0.06 (s, 9H), −0.18 (s, 9H); ^{13}C NMR (126 MHz, $CDCl_3$) δ 174.0, 159.0, 157.7, 142.5, 137.1, 130.9, 129.1 (2C), 128.7 (br, 2C), 127.1 (2C), 124.1, 113.8 (br, 2C), 82.2, 71.2, 69.3, 55.4, 50.4, 28.0 (3C), 21.5, 12.1, –0.3 (3C), –0.9 (3C); HRMS–ESI (positive mode; M + Na) *m*/*z* 652.2377, calcd for $C_{31}H_{47}NNaO_5SSi_3$, 652.2381.

(3*S*,3a*S*)-*tert*-Butyl 3-(3-chlorophenyl)-2-tosyl-5,5-bis(trimethylsilyl)-1,2,3,3a,4,5-hexahydrosilolo[3,4-*c*]pyrrole-3a-carboxylate (*trans* 2e): The reaction was performed in a similar manner to the preparation of **2a**, starting with **1e** (94 mg, 0.205 mmol) with $(Me_3Si)_3SiH$ (0.06 mL, 0.195 mmol) and Et_3B (1.0 M in hexane, 0.60 mL, 0.60 mmol) giving **2e** in 42% yield (54.2 mg, 0.0855 mmol). Further chromatographic purification gave *trans* **2e**. Colorless oil; $[\alpha]_D$ −54.1 (*c* 0.68, $CHCl_3$); ^1H NMR (500 MHz, $CDCl_3$) δ 7.33 (d, *J* = 8.0 Hz, 2H), 7.11 (d, *J* = 6.8 Hz, 1H), 7.06 (d, *J* = 8.1 Hz, 2H), 7.02–6.08 (m, 3H), 5.89 (s, 1H), 5.18 (s, 1H), 4.43 (d, *J* = 12.7 Hz, 1H), 3.96 (d, *J* = 14.1 Hz, 1H), 2.33 (s, 3H), 1.51 (s, 9H), 1.17 (d, *J* = 14.9 Hz, 1H), 0.44 (d, *J* = 14.9 Hz, 1H), 0.07 (s, 9H), −0.19 (s, 9H); ^{13}C NMR (126 MHz, $CDCl_3$) δ 173.5, 156.9, 143.1, 140.6 (br), 136.8, 134.4 (br), 129.5, 129.3 (br, 3C), 127.7, 126.8 (br, 3C), 124.9, 82.5, 71.0, 69.0, 50.5, 28.0 (3C), 21.5, 12.1, −0.3 (3C), −1.1 (3C); HRMS–ESI (positive mode; M + Na) *m*/*z* 656.1901, calcd for $C_{30}H_{44}ClNNaO_4SSi_3$, 656.1885.

(3*S*,3a*S*)-*tert*-Butyl 3-(4-chlorophenyl)-2-tosyl-5,5-bis(trimethylsilyl)-1,2,3,3a,4,5-hexahydrosilolo[3,4-*c*]pyrrole-3a-carboxylate (*trans* 2f). The reaction was performed in a similar manner to the preparation of **2a**, starting with **1f** (92.4 mg, 0.201 mmol) with $(Me_3Si)_3SiH$ (0.07 mL, 0.228 mmol) and Et_3B (1.0 M in hexane, 0.60 mL, 0.60 mmol) giving **2f** in 51% yield (65.4 mg, 0.103 mmol). Further chromatographic purification gave *trans* **2f**. Colorless oil; $[\alpha]_D$ −51.5 (c 1.04, $CHCl_3$); the enantiomeric purity was determined by HPLC analysis, t_R 8.02 min (major), t_R 9.79 min (minor) [CHIRALPAK ID (0.46 cm × 25 cm) (from Daicel Chemical Ind., Ltd.) hexane/iPrOH, 95/5, 40 °C, 1.0 mL/min] as 90% ee; ^1H NMR (500 MHz, $CDCl_3$) δ 7.34 (d, *J* = 7.3 Hz, 2H), 7.07 (d, *J* = 8.2 Hz, 1H), 7.18–6.42 (br, 4H), 5.87 (s, 1H), 5.18 (s, 1H), 4.39 (dd, *J* = 13.3, 1.8 Hz, 1H), 3.95 (d, *J* = 13.1 Hz, 1H), 2.34 (s, 3H), 1.48 (s, 9H), 1.14 (dd, *J* = 15.0, 1.0 Hz, 1H), 0.46 (dd, *J* = 14.8, 1.0 Hz, 1H), 0.06 (s,

9H), −0.19 (s, 9H); ^{13}C NMR (126 MHz, $CDCl_3$) δ 173.6, 157.0, 143.0, 137.4, 136.9, 133.4, 129.3 (2C), 128.4 (br, 4C), 127.0 (2C), 124.8, 82.4, 71.0, 68.9, 50.4, 28.0 (3C), 21.5, 12.2, −0.3 (3C), −1.0 (3C); HRMS–ESI (positive mode; M + Na) *m*/*z* 656.1898, calcd for $C_{30}H_{44}ClNNaO_4SSi_3$, 656.1885.

(3*S*,3a*S*)-*tert*-Butyl 3-(4-fluorophenyl)-2-tosyl-5,5-bis(trimethylsilyl)-1,2,3,3a,4,5-hexahydrosilolo[3,4-*c*]pyrrole-3a-carboxylate (*trans* 2g). The reaction was performed in a similar manner to the preparation of **2a**, starting with **1g** (87.8 mg, 0.198 mmol) with $(Me_3Si)_3SiH$ (0.07 mL, 0.228 mmol) and Et_3B (1.0 M in hexane, 0.60 mL, 0.60 mmol) giving **2g** in 61% yield (74.6 mg, 0.121 mmol). Further chromatographic purification gave *trans* **2g**. Colorless oil; $[\alpha]_D$ −21.2 (*c* 0.52, $CHCl_3$); the enantiomeric purity was determined by HPLC analysis, t_R 8.1 min (major), t_R 9.9 min (minor) [CHIRALPAK ID (0.46 cm × 25 cm) (from Daicel Chemical Ind., Ltd.) hexane/iPrOH, 95/5, 40 °C, 1.0 mL/min] as 97% ee; ^{1}H NMR (500 MHz, $CDCl_3$) δ 7.33 (d, *J* = 8.3 Hz, 2H), 7.06 (d, *J* = 8.6 Hz, 2H), 6.98–6.62 (m, 4H), 5.87 (s, 1H), 5.20 (s, 1H), 4.40 (dd, *J* = 12.9, 2.2 Hz, 1H), 3.94 (dd, *J* = 12.9, 1.3 Hz, 1H), 2.33 (s, 3H), 1.49 (s, 9H), 1.14 (d, *J* = 14.9 Hz, 1H), 0.46 (d, *J* = 14.9 Hz, 1H), 0.07 (s, 9H), −0.19 (s, 9H); ^{13}C NMR (126 MHz, $CDCl_3$) δ 173.7, 162.2 (d, *J* = 245.9 Hz), 157.2, 142.9, 137.0, 134.7 (2C, d, *J* = 3.3 Hz), 129.2 (2C), 128.0, 127.0 (2C), 124.6, 115.3 (Br, 2C), 82.4, 71.1, 68.9, 50.4, 28.0 (3C), 21.5, 12.2, −0.3 (3C), −1.0 (3C); HRMS–ESI (positive mode; M + Na) *m*/*z* 640.2182, calcd for $C_{30}H_{44}FNNaO_4SSi_3$, 640.2181.

(3*S*,3a*S*)-*tert*-Butyl 2-tosyl-3-(4-(trifluoromethyl)phenyl)-5,5-bis(trimethylsilyl)-1,2,3,3a,4,5-hexahydrosilolo[3,4-*c*]pyrrole-3a-carboxylate (*trans* 2h). The reaction was performed in a similar manner to the preparation of **2a**, starting with **1h** (95 mg, 0.201 mmol) with $(Me_3Si)_3SiH$ (0.07 mL, 0.228 mmol) and Et_3B (1.0 M in hexane, 0.60 mL, 0.60 mmol) giving **2h** in 61% yield (70.8 mg, 0.106 mmol). Further

chromatographic purification gave *trans* **2h**. Colorless oil; $[\alpha]_D$ −33.6 (*c* 0.28, $CHCl_3$); the enantiomeric purity was determined by HPLC analysis, t_R 7.0 min (major), t_R 8.4 min (minor) [CHIRALPAK ID (0.46 cm × 25 cm) (from Daicel Chemical Ind., Ltd.) hexane/iPrOH, 95/5, 40 °C, 0.9 mL/min] as 68% ee; ^1H NMR (500 MHz, $CDCl_3$) δ 7.70–7.27 (m, 4H), 7.33 (d, J = 8.4 Hz, 2H), 7.03 (dd, J = 7.8, 1.1 Hz, 2H), 5.90 (s, 1H), 5.25 (s, 1H), 4.43 (d, J = 11.3 Hz, 1H), 4.01 (d, J = 13.0 Hz, 1H), 2.31 (s, 3H), 1.49 (s, 9H), 1.15 (d, J = 15.0 Hz, 1H), 0.38 (d, J = 15.6 Hz, 1H), 0.07 (s, 9H), −0.23 (s, 9H); ^{13}C NMR (126 MHz, $CDCl_3$) δ 173.4, 156.7, 143.1, 142.8, 136.9, 130.0, 129.7 (q, J = 32.4 Hz), 129.3 (2C), 127.5 (q, J = 134.2 Hz), 126.9 (2C), 125.2 (br, 2C), 125.1, 124.5, 82.6, 71.0, 68.9, 50.6, 28.0 (3C), 21.4, 12.2, −0.3 (3C), −1.1 (3C); HRMS–ESI (positive mode; M + Na) *m*/*z* 690.2168, calcd for $C_{31}H_{44}F_3NNaO_4SSi_3$, 690.2149.

(3*R*,3a*S*)-*tert*-Butyl 3-(thiophen-2-yl)-2-tosyl-5,5-bis(trimethylsilyl)-1,2,3,3a,4,5-hexahydrosilolo[3,4-*c*]pyrrole-3a-carboxylate (*trans* 2i). The reaction was performed in a similar manner to the preparation of **2a**, starting with **1i** (98.0 mg, 0.23 mmol) with $(Me_3Si)_3SiH$ (0.06 mL, 0.195 mmol) and Et_3B (1.0 M in hexane, 0.60 mL, 0.60 mmol) giving **2i** in 48% yield (67.3 mg, 0.111 mmol). Further chromatographic purification gave *trans* **2i**. White solid; mp 145–146 °C; $[\alpha]_D$ −84.9 (*c* 0.68, $CHCl_3$); the enantiomeric purity was determined by HPLC analysis, t_R 8.50 min (major), t_R 12.0 min (minor) [CHIRALPAK ID (0.46 cm × 25 cm) (from Daicel Chemical Ind., Ltd.) hexane/iPrOH, 95/5, 40 °C, 1.0 mL/min] as 98% ee; ^1H NMR (500 MHz, $CDCl_3$) δ 7.30 (d, J = 8.2 Hz, 2H), 7.07–6.98 (m, 3H), 6.82 – 6.77 (m, 1H), 6.69 (d, J = 3.2 Hz, 1H), 5.91 (s, 1H), 5.54 (s, 1H), 4.36 (d, J = 13.1 Hz, 1H), 3.77 (d, J = 13.3 Hz, 1H), 2.31 (s, 3H), 1.53 (s, 9H), 1.16 (d, J = 15.0 Hz, 1H), 0.69 (d, J = 15.0 Hz, 1H), 0.08 (s, 9H), −0.12 (s, 9H); ^{13}C NMR (126 MHz, $CDCl_3$) δ 173.6, 157.1, 142.5, 141.2, 136.8,

129.1 (2C), 127.0, 126.8 (2C), 126.4, 125.6, 125.2, 82.5, 71.5, 65.7, 49.3, 28.0 (3C), 21.5, 11.6, −0.2 (3C), −0.6 (3C); HRMS–ESI (positive mode; M + Na) *m*/*z* 628.1822, calcd for $C_{28}H_{43}NNaO_4S_2Si_3$, 628.1839.

(3*S*,3a*S*)-*tert*-Butyl 3-(naphthalen-2-yl)-2-tosyl-5,5-bis(trimethylsilyl)-1,2,3,3a,4,5-hexahydrosilolo[3,4-*c*]pyrrole-3a-carboxylate (*trans* 2j). The reaction was performed in a similar manner to the preparation of **2a**, starting with **1j** (95.0 mg, 0.215 mmol) with $(Me_3Si)_3SiH$ (0.06 mL, 0.195 mmol) and Et_3B (1.0 M in hexane, 0.60 mL, 0.60 mmol) giving **2j** in 51% yield (71.0 mg, 0.109 mmol). Further chromatographic purification gave *trans* **2j**. White solid; mp 150–151 °C; $[\alpha]_D$ −100.5 (*c* 0.68, $CHCl_3$); the enantiomeric purity was determined by HPLC analysis, t_R 10.5 min (major), t_R 12.5 min (minor) [CHIRALPAK ID (0.46 cm × 25 cm) (from Daicel Chemical Ind., Ltd.) hexane/iPrOH, 95/5, 40 °C, 1.0 mL/min] as 99% ee; ^{1}H NMR (500 MHz, $CDCl_3$) δ 7.80–7.68 (m, 1H), 7.48–7.34 (m, 2H), 7.99–7.04 (br, 6H), 6.81 (d, *J* = 8.0 Hz, 2H), 5.91 (s, 1H), 5.37 (s, 1H), 4.50 (d, *J* = 13.0 Hz, 1H), 4.06 (d, *J* = 12.9 Hz, 1H), 2.16 (s, 3H), 1.55 (s, 9H), 1.19 (d, *J* = 14.9 Hz, 1H), 0.52 (d, *J* = 14.9 Hz, 1H), 0.06 (s, 9H), −0.15 to −0.60 (br, 9H); ^{13}C NMR (126 MHz, $CDCl_3$) δ 173.9, 157.6 (br), 150.3 (br), 142.6, 137.0, 135.6 (br), 133.1, 132.8 (br), 129.0 (2C), 128.1, 127.5 (2C), 126.9, 125.9 (br, 3C), 124.4 (br, 2C), 82.4, 71.2, 69.8, 50.7, 28.1 (3C), 21.3, 12.3, -0.3 (3C), -1.1 (3C); HRMS–ESI (positive mode; M + Na) *m*/*z* 672.2453, calcd for $C_{34}H_{47}NNaO_4SSi_3$, 672.2431.

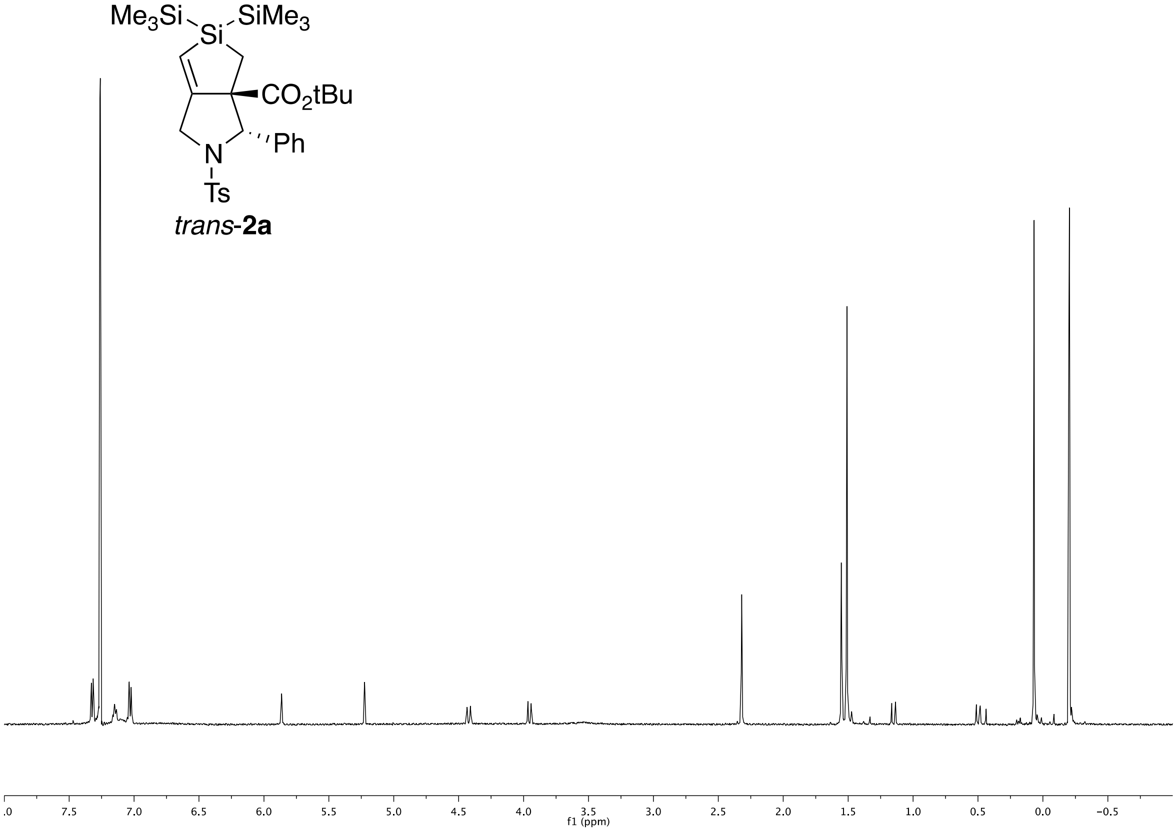
Me3Si
SiMe3
Si
CO2tBu
Ph
N
Ts
trans-**2a**
f1 (ppm)

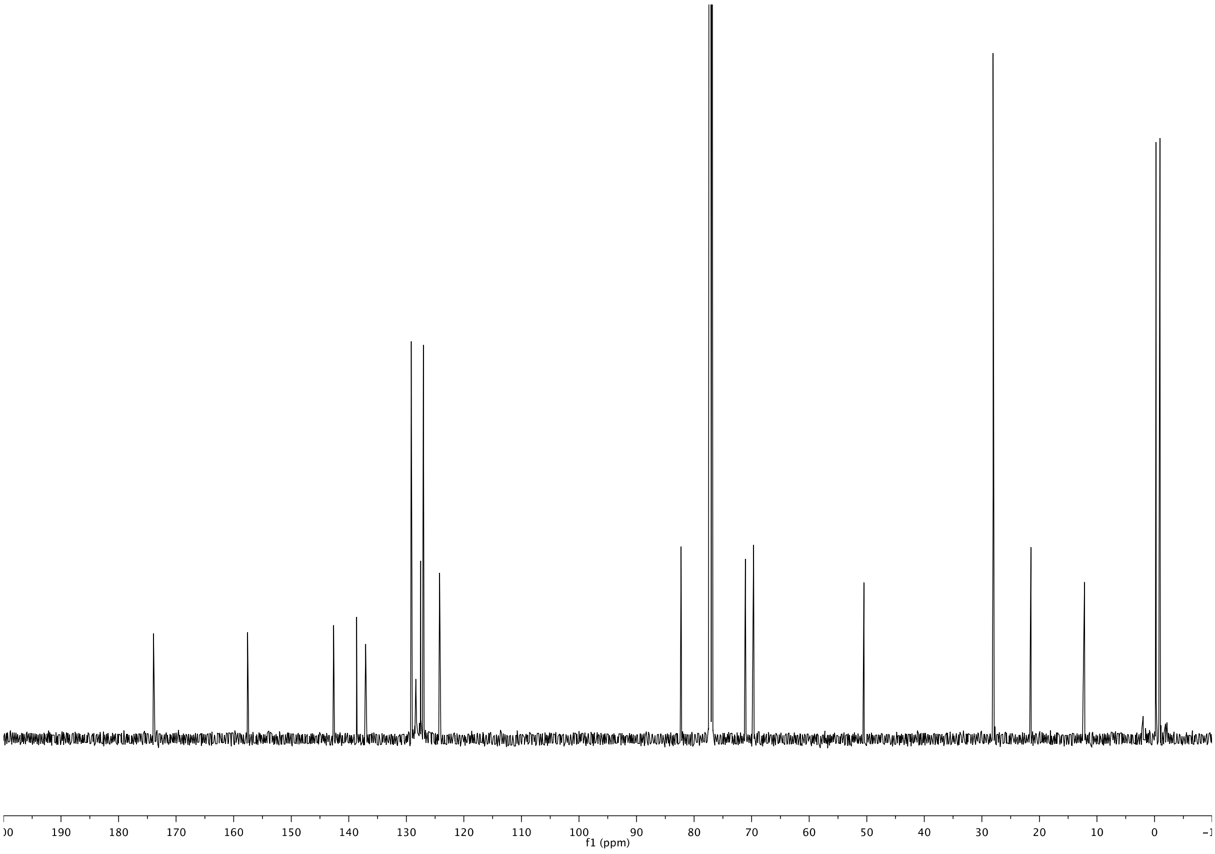
f1 (ppm)

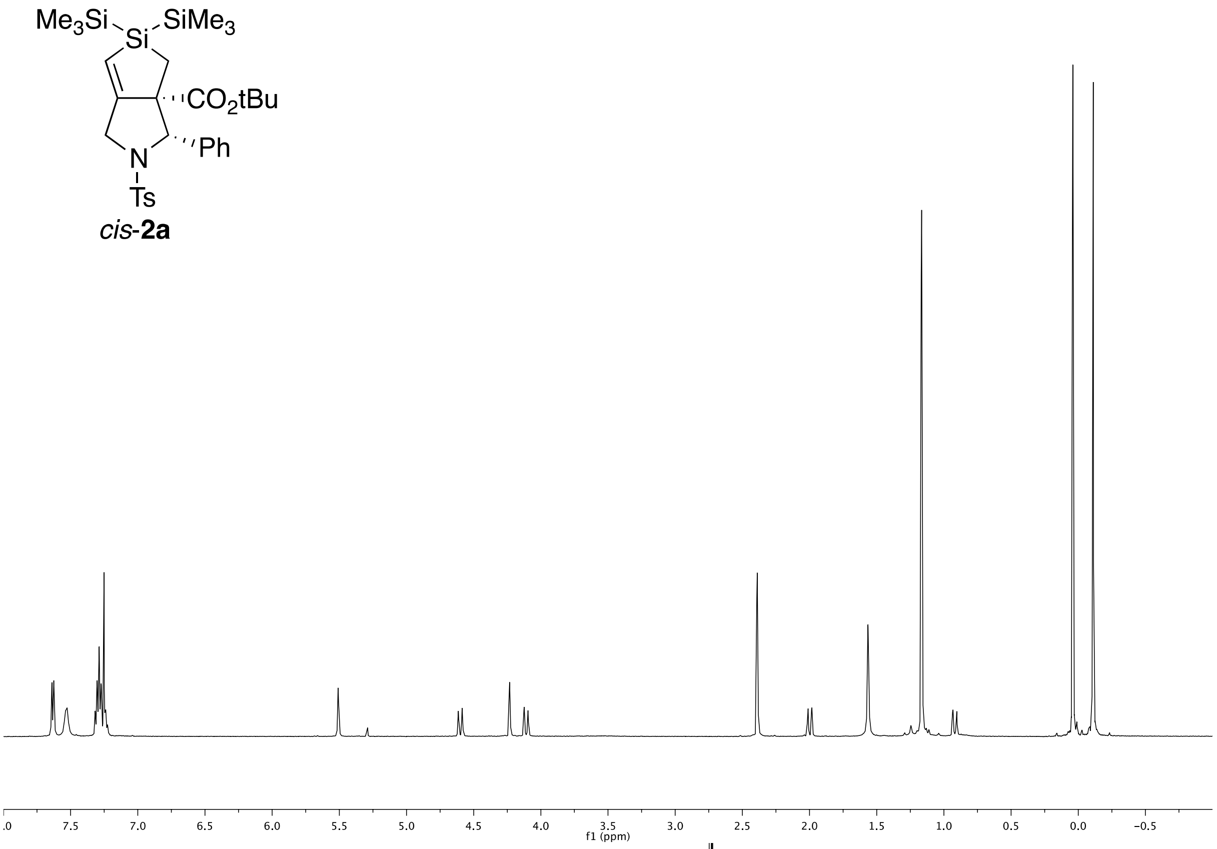

cis-**2a**

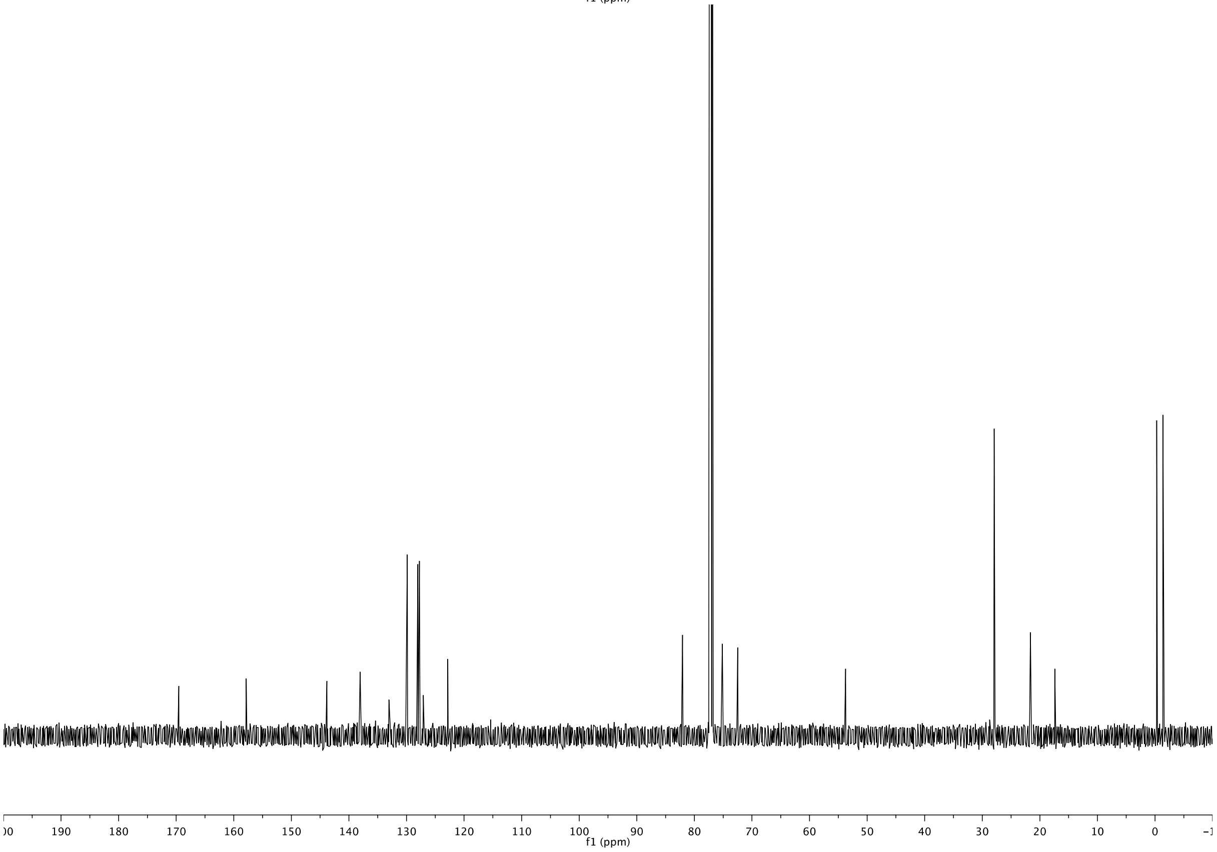

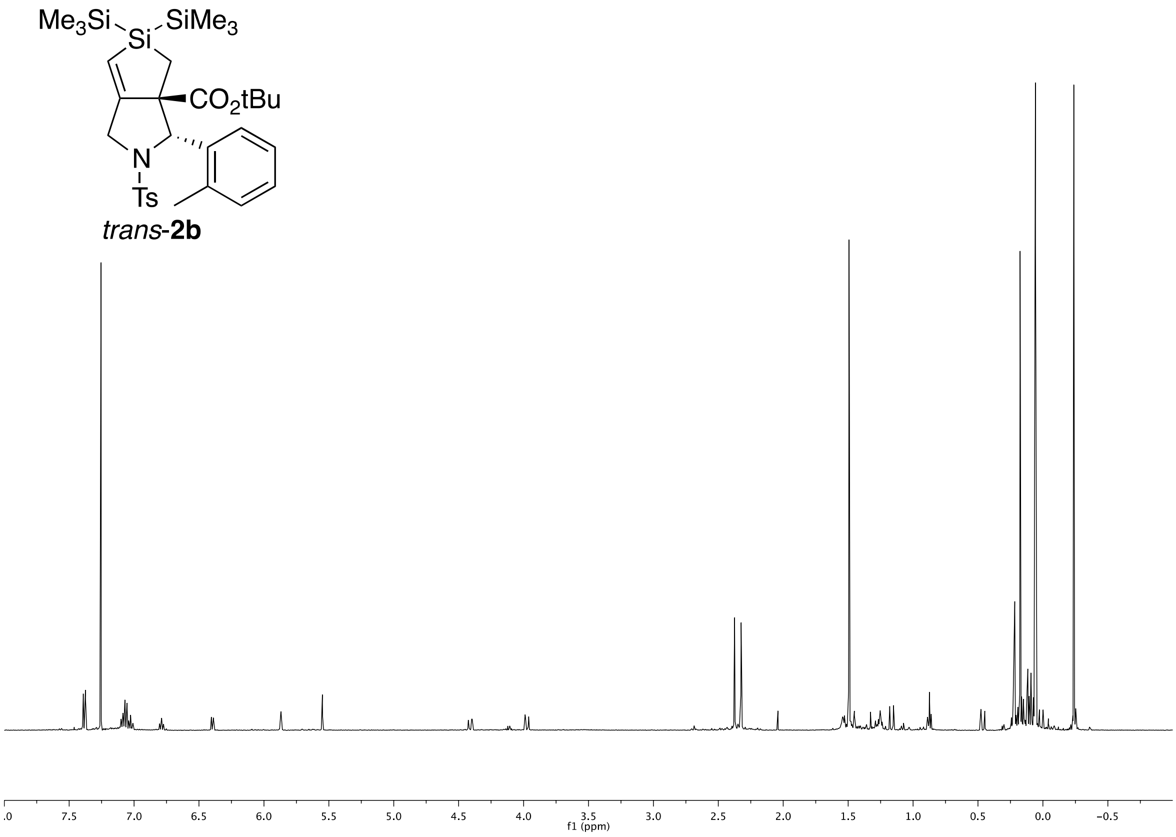

trans-**2b**

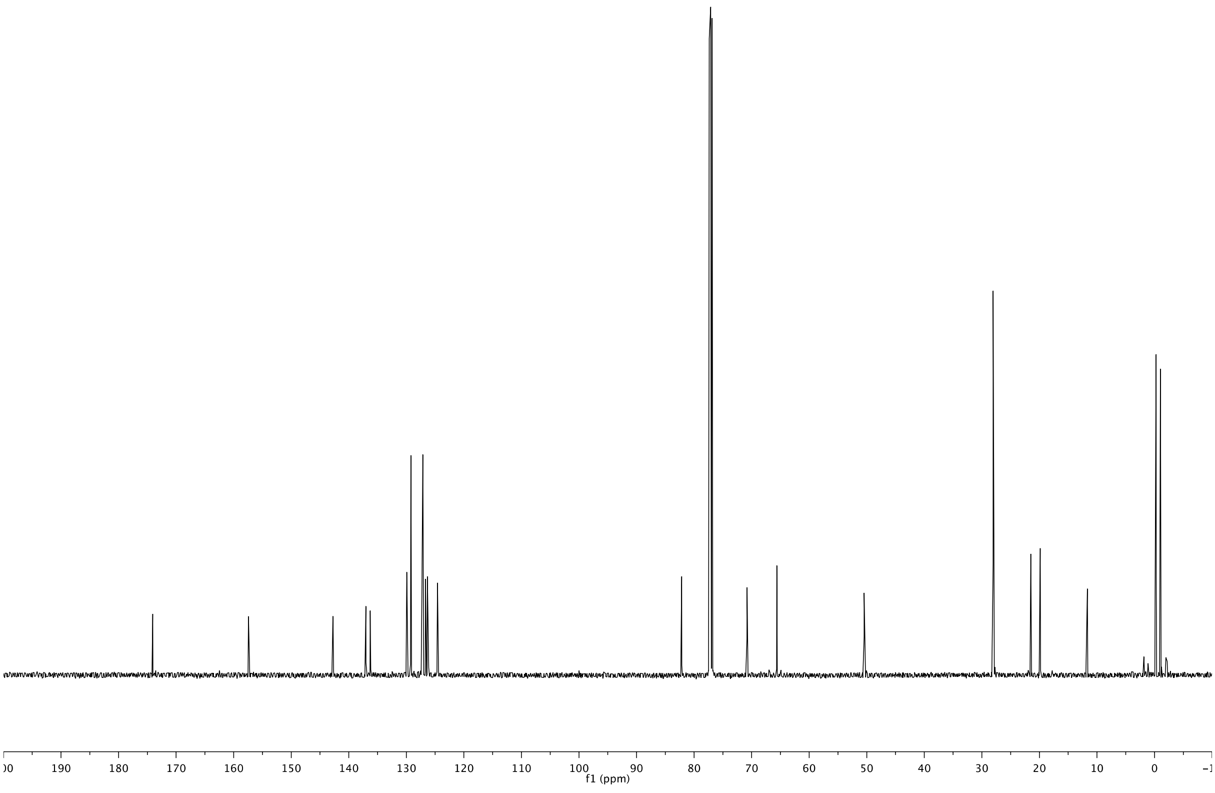

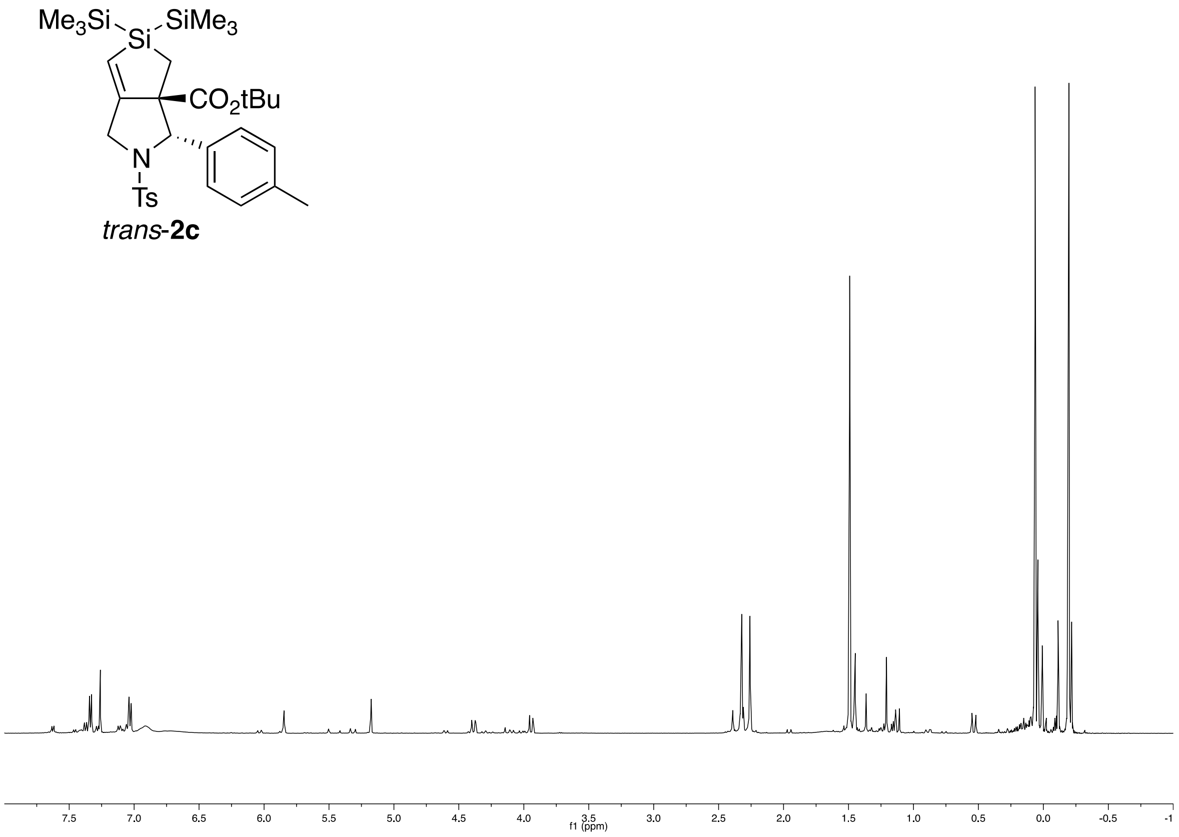

Me3Si
SiMe3
Si
CO2tBu
N
Ts
trans-2c
f1 (ppm)

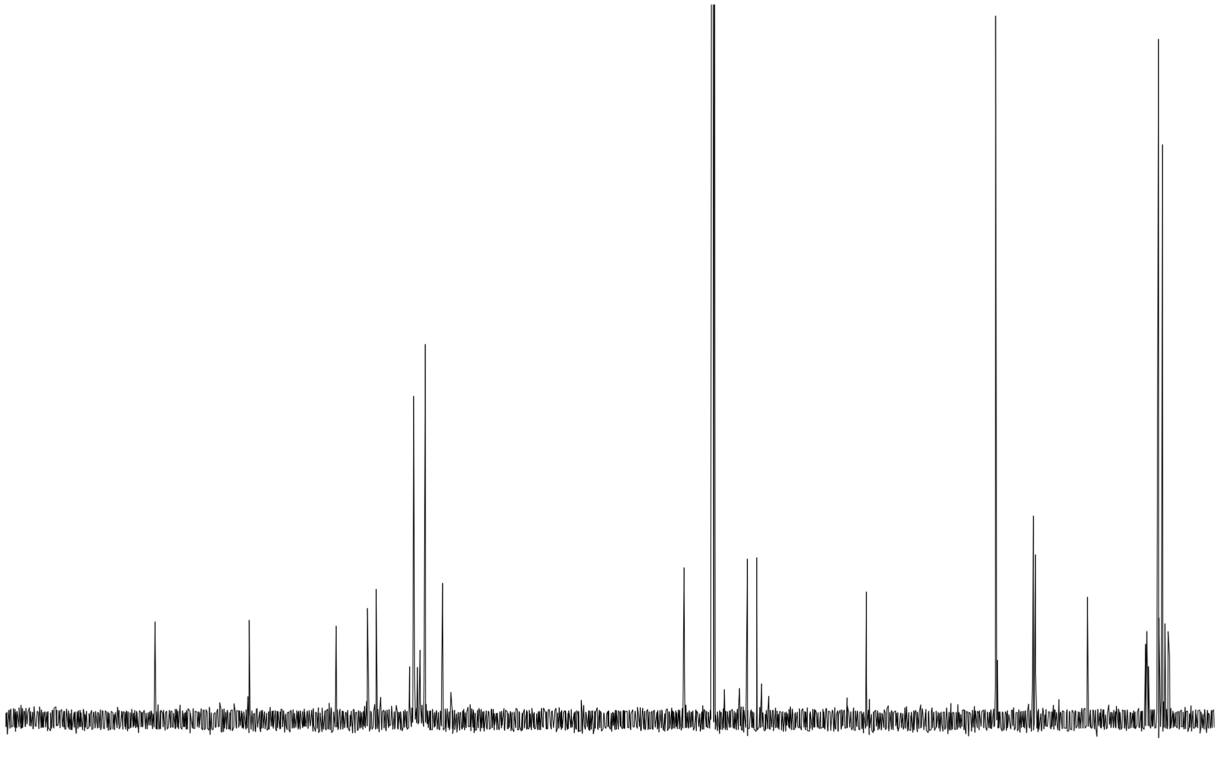

f1 (ppm)

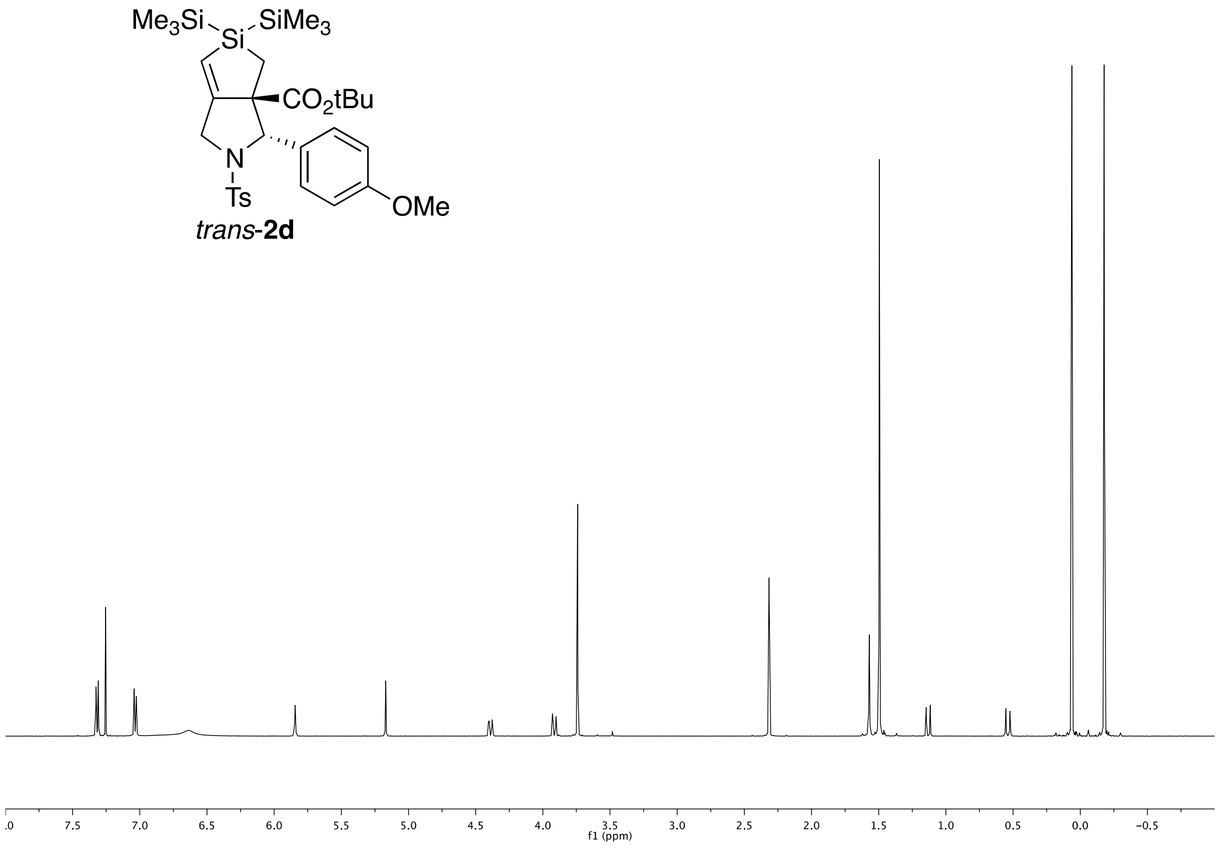

trans-**2d**

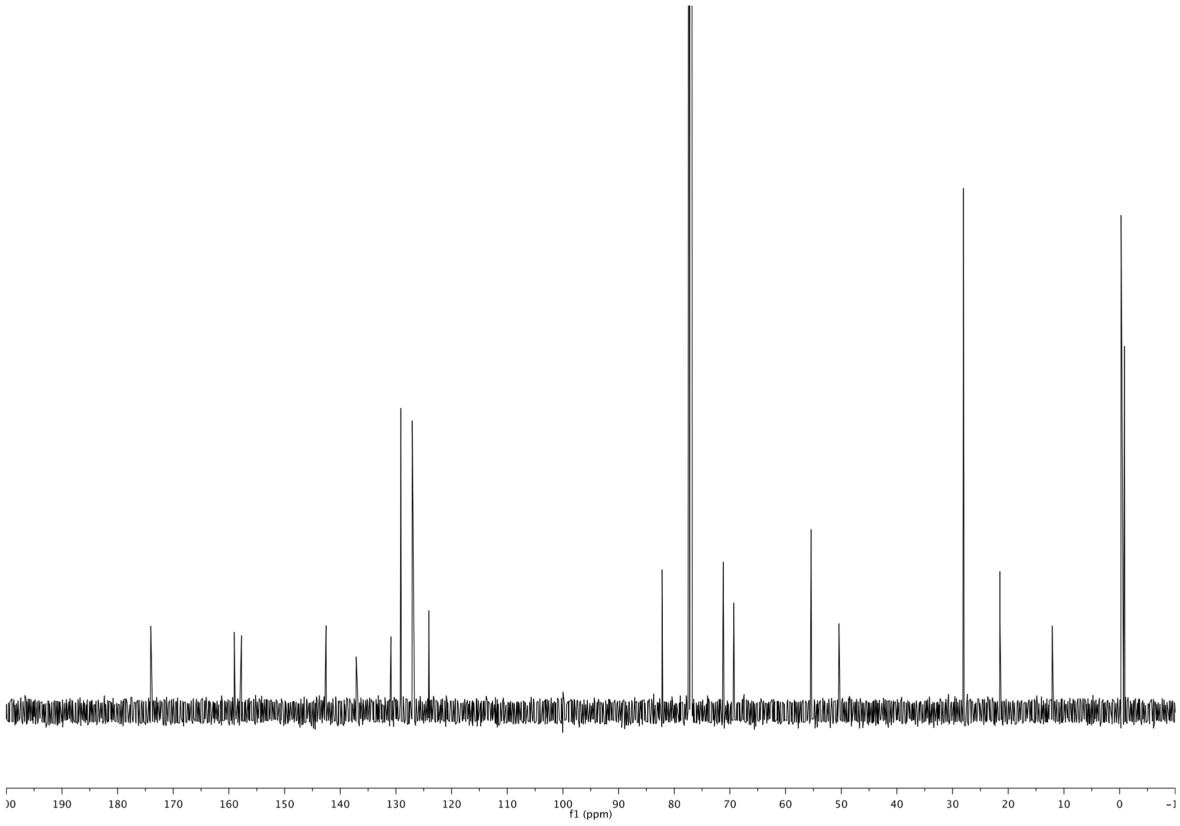

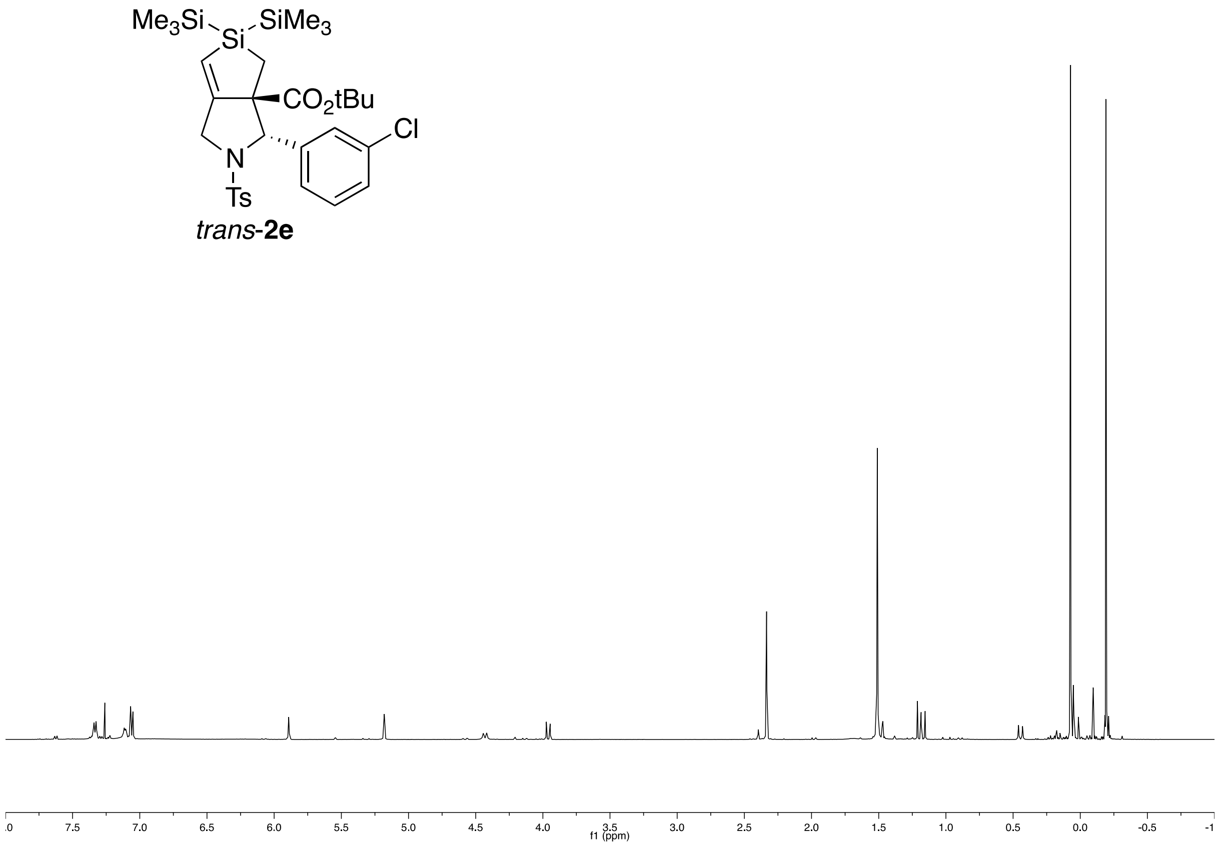
Me3Si
SiMe3
Si
CO2tBu
Cl
N
Ts
trans-2e
f1 (ppm)

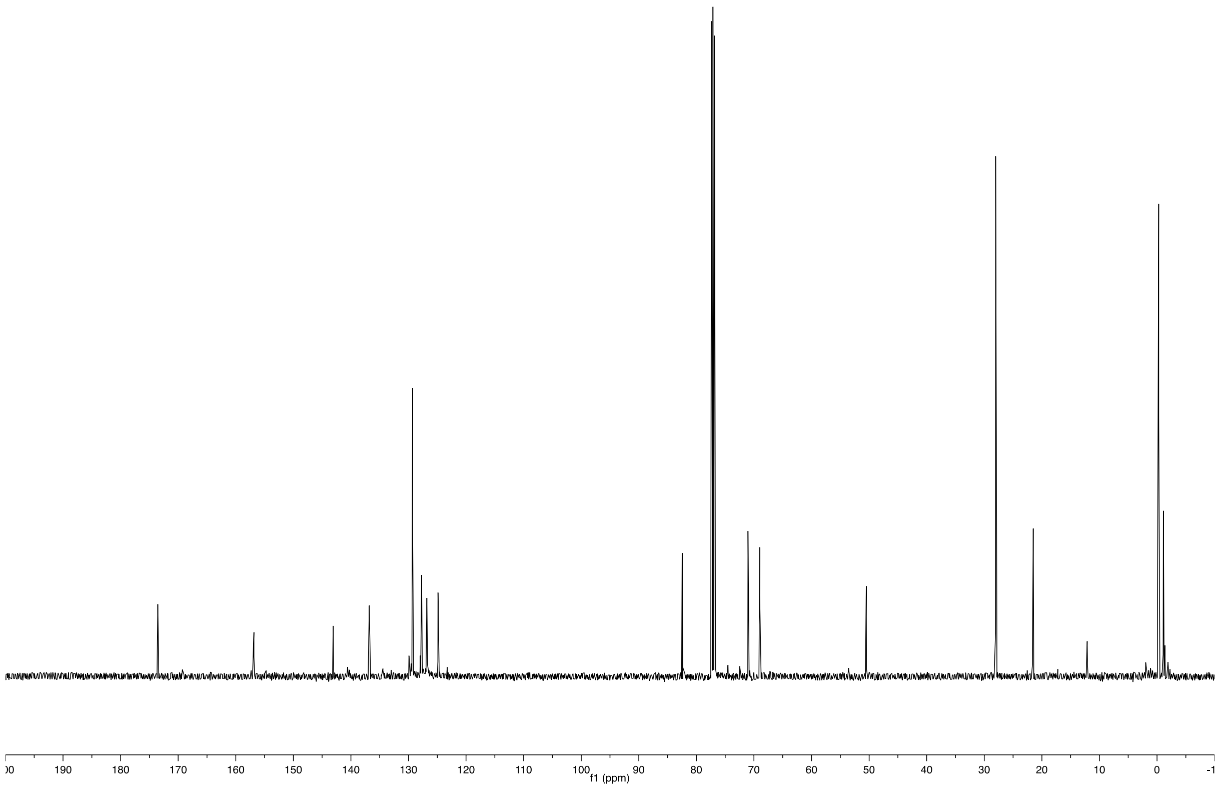
f1 (ppm)

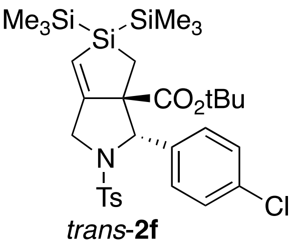

trans-**2f**

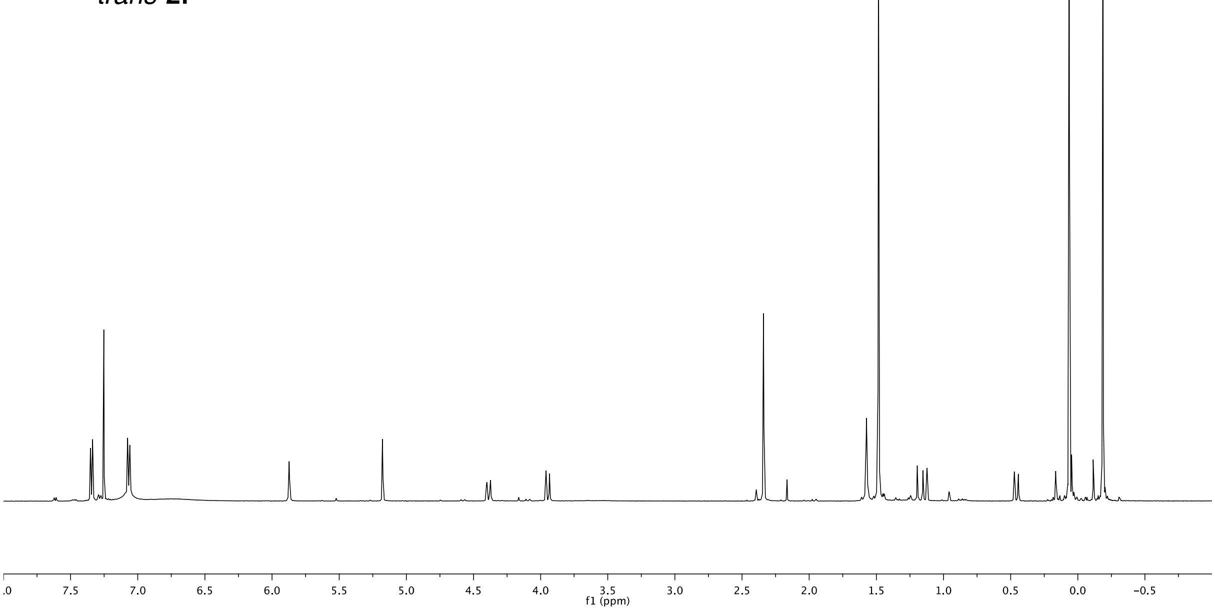

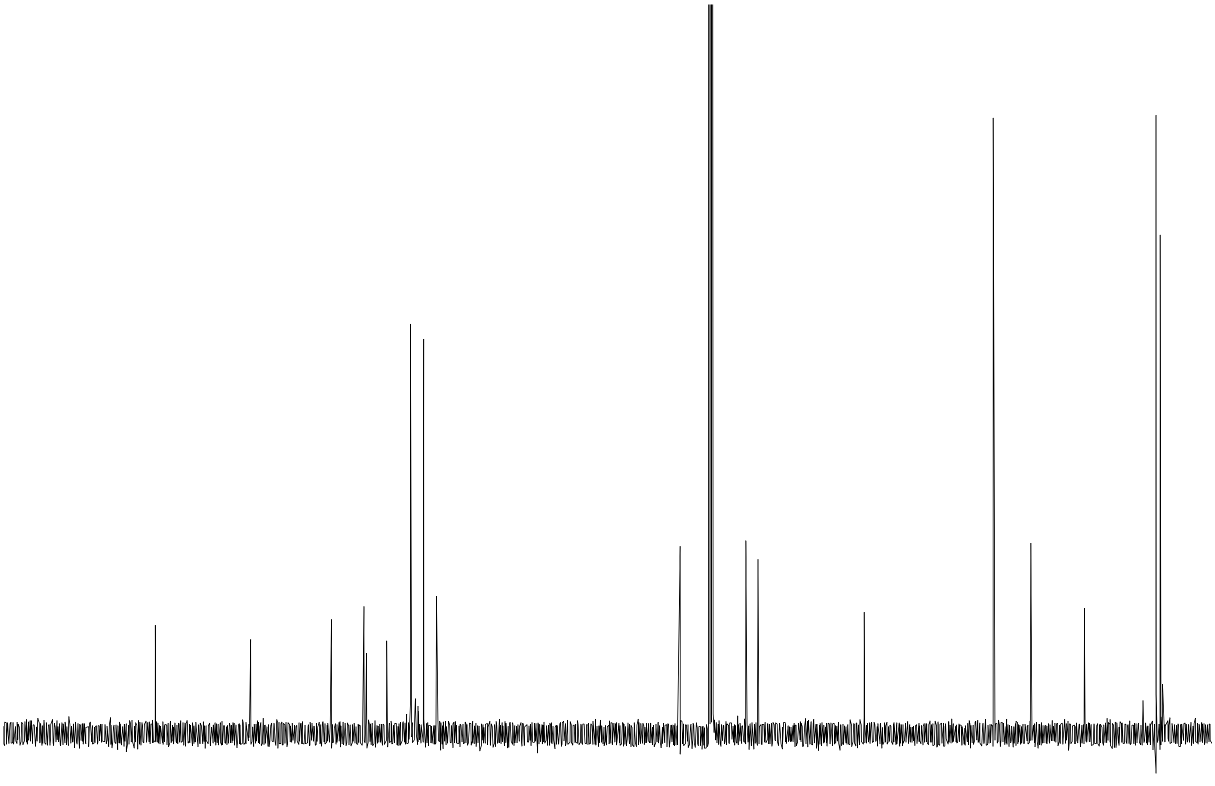

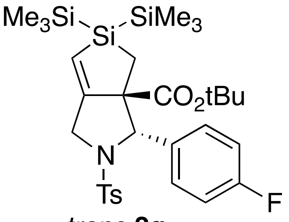

trans-**2g**

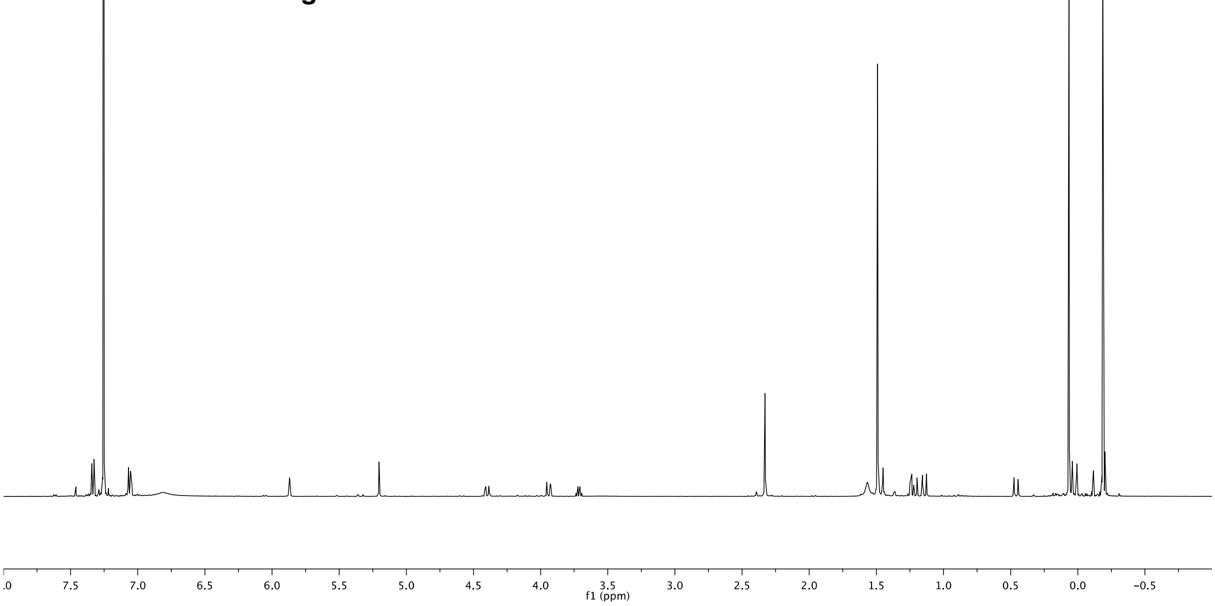

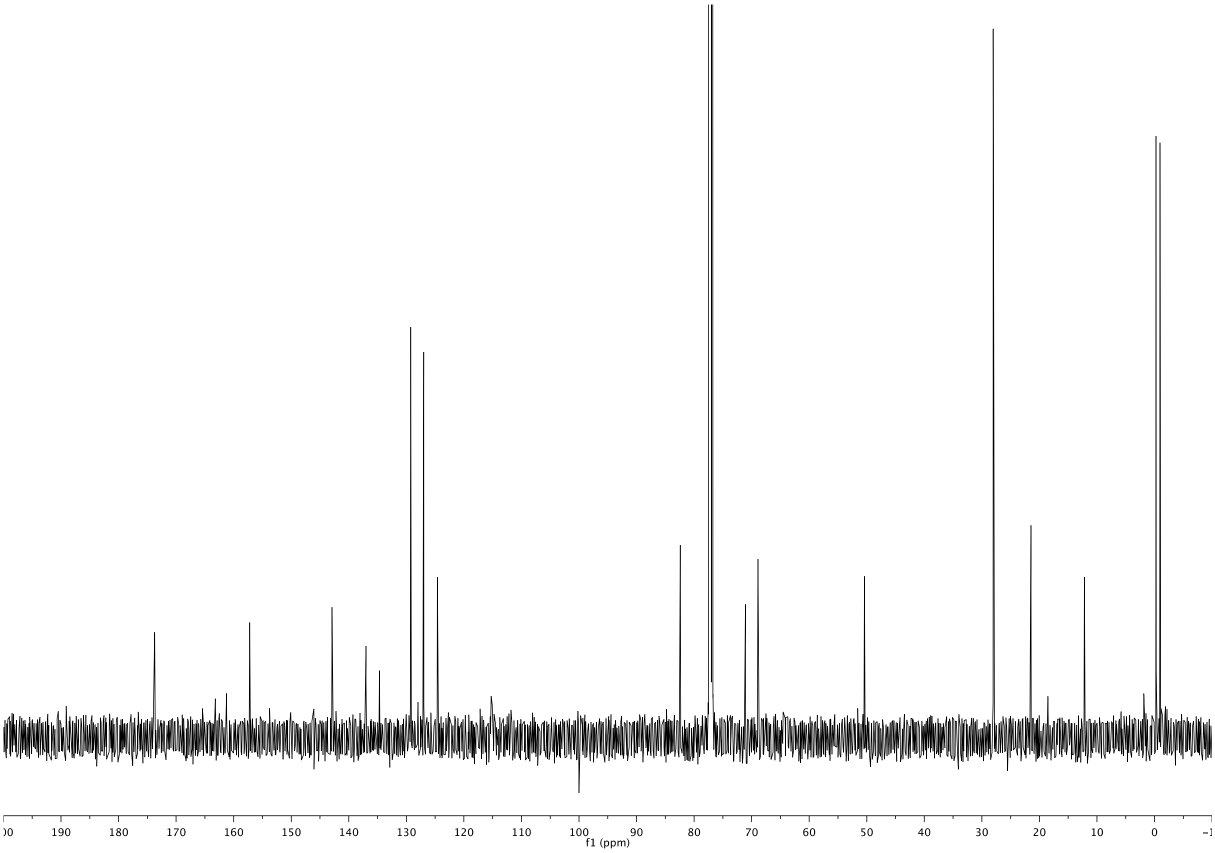

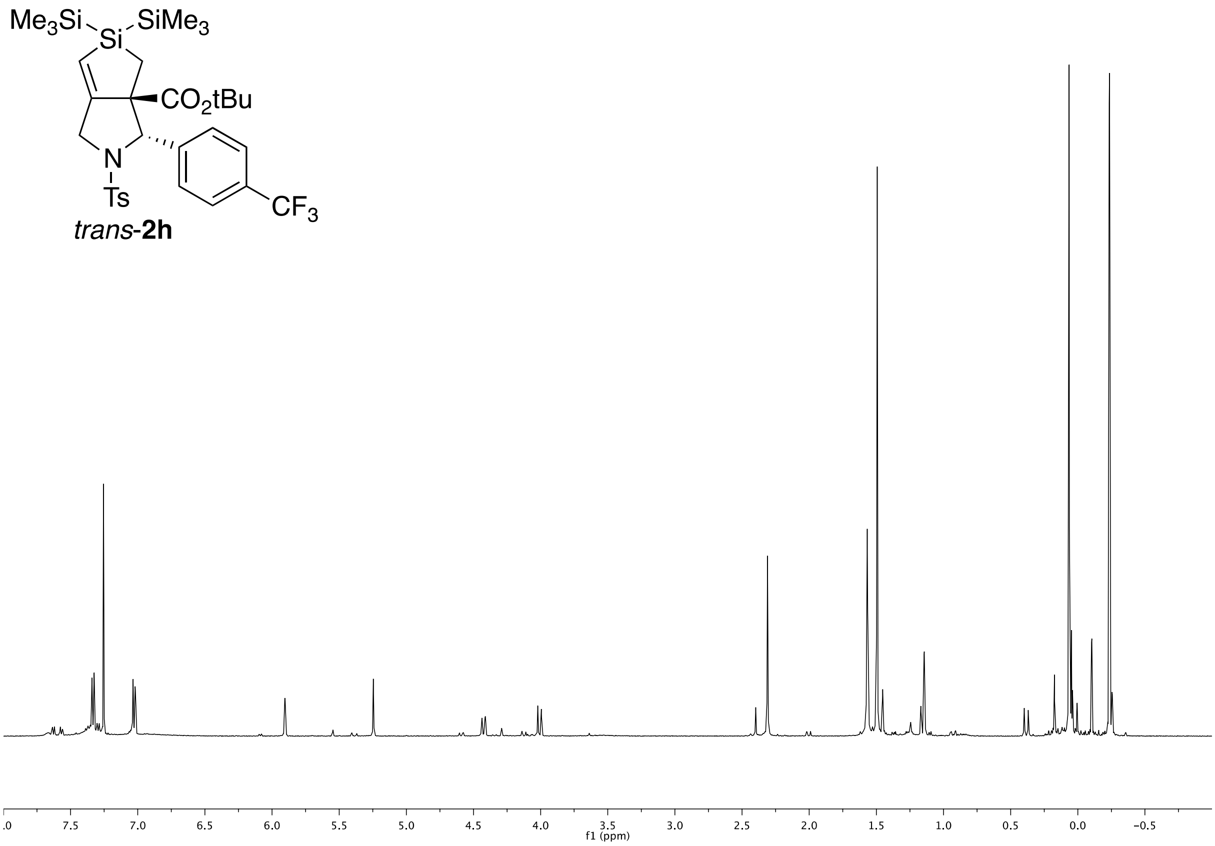

trans-**2h**

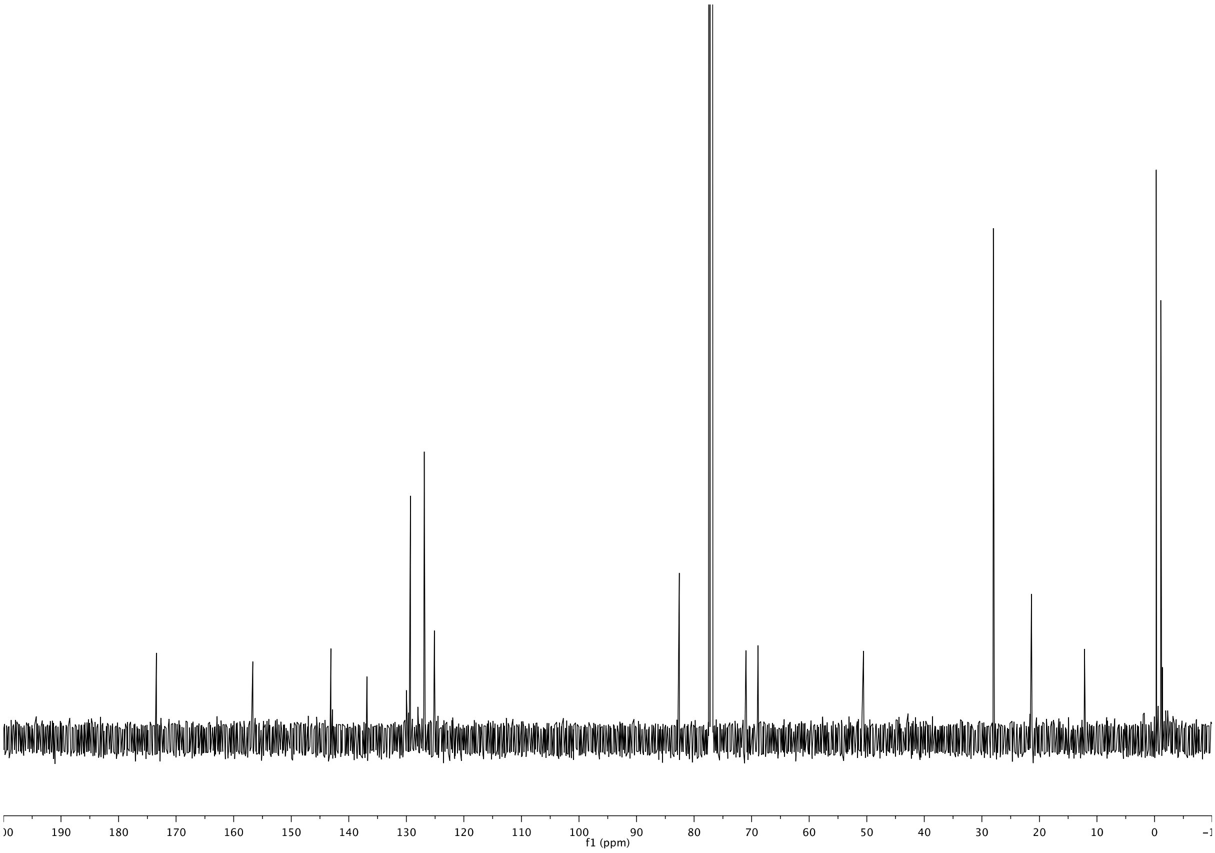

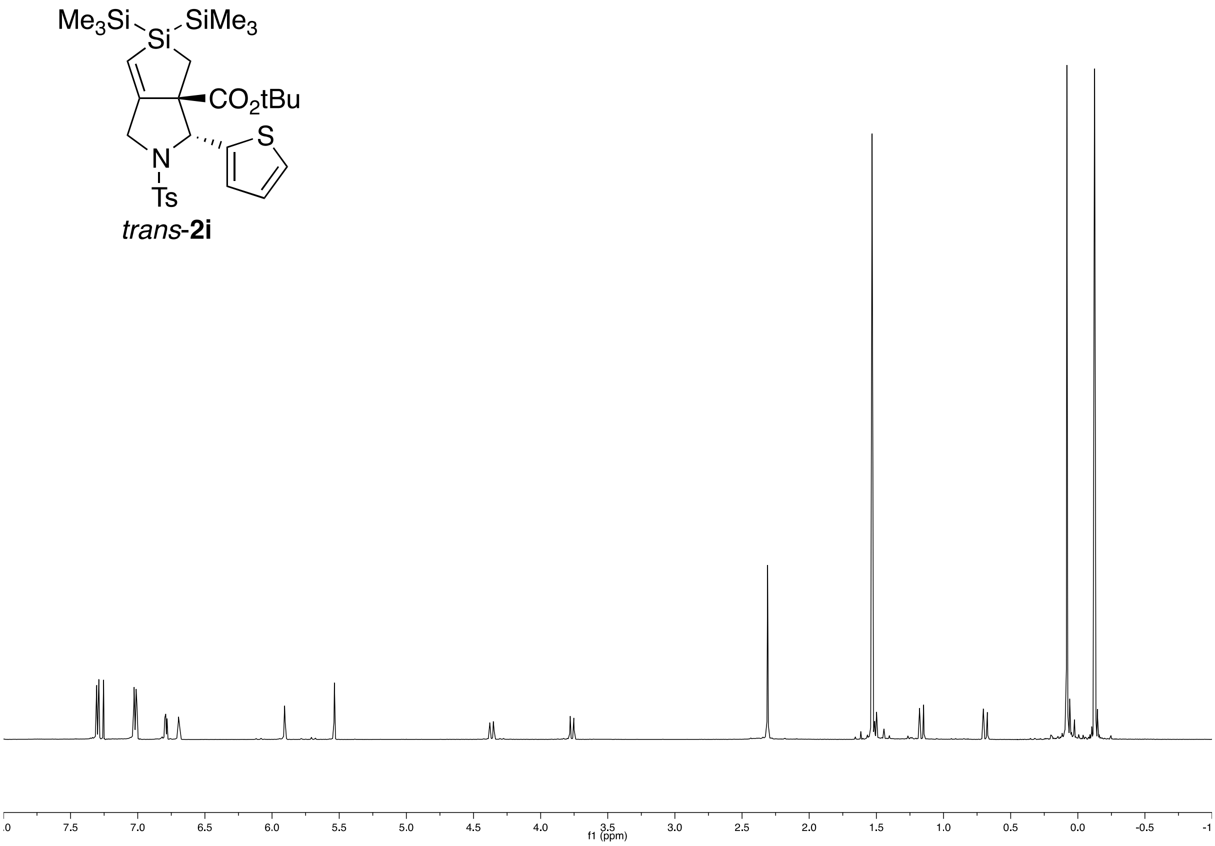

trans-**2i**

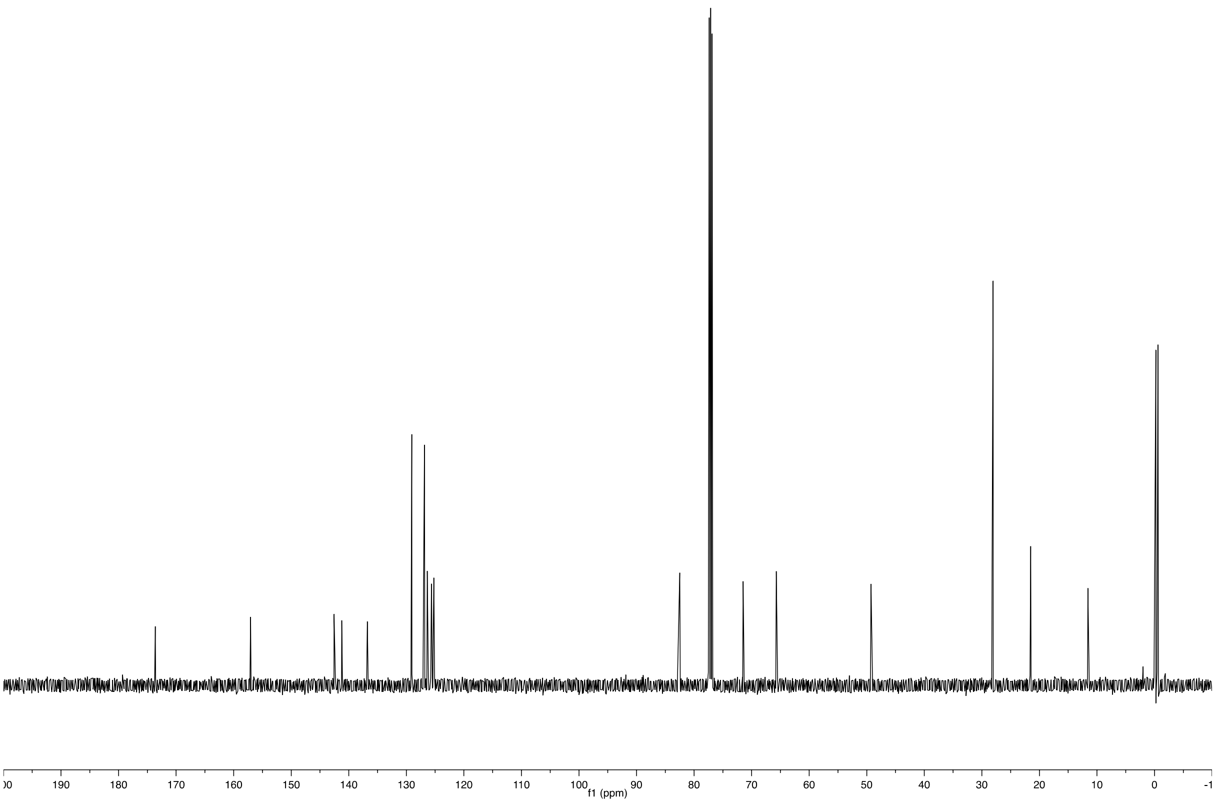

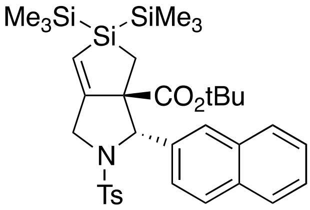

trans-**2j**

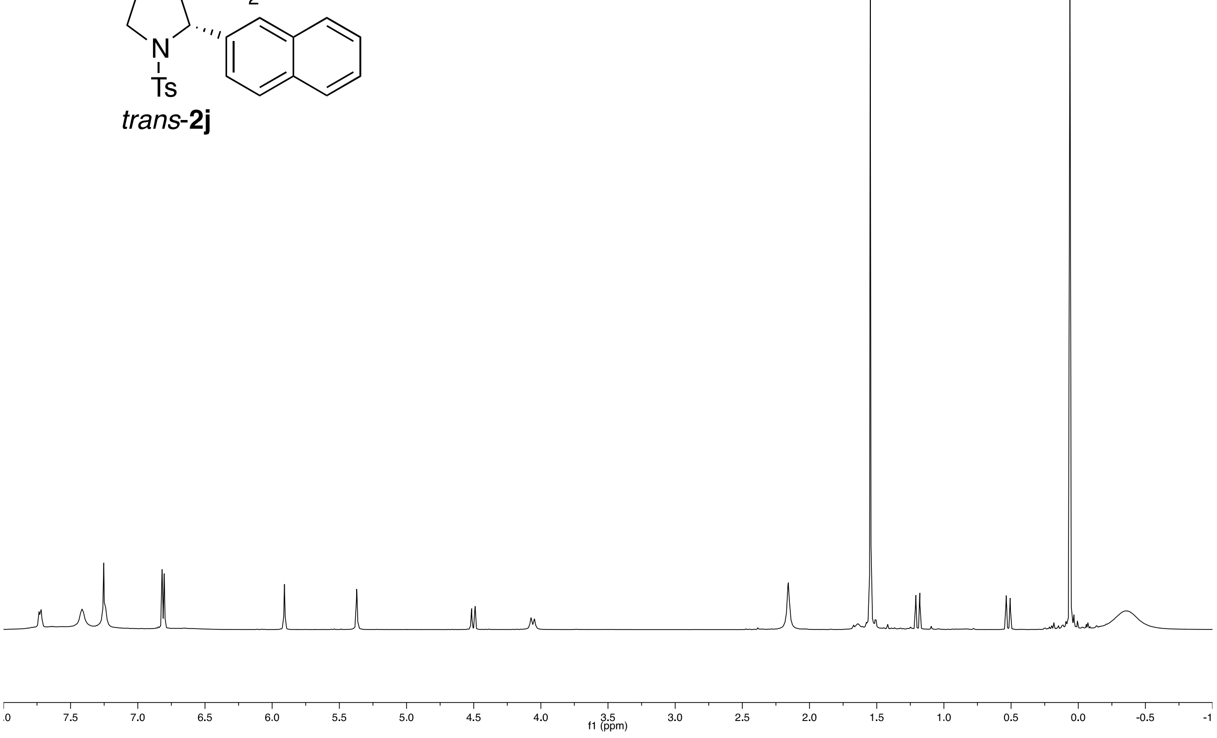

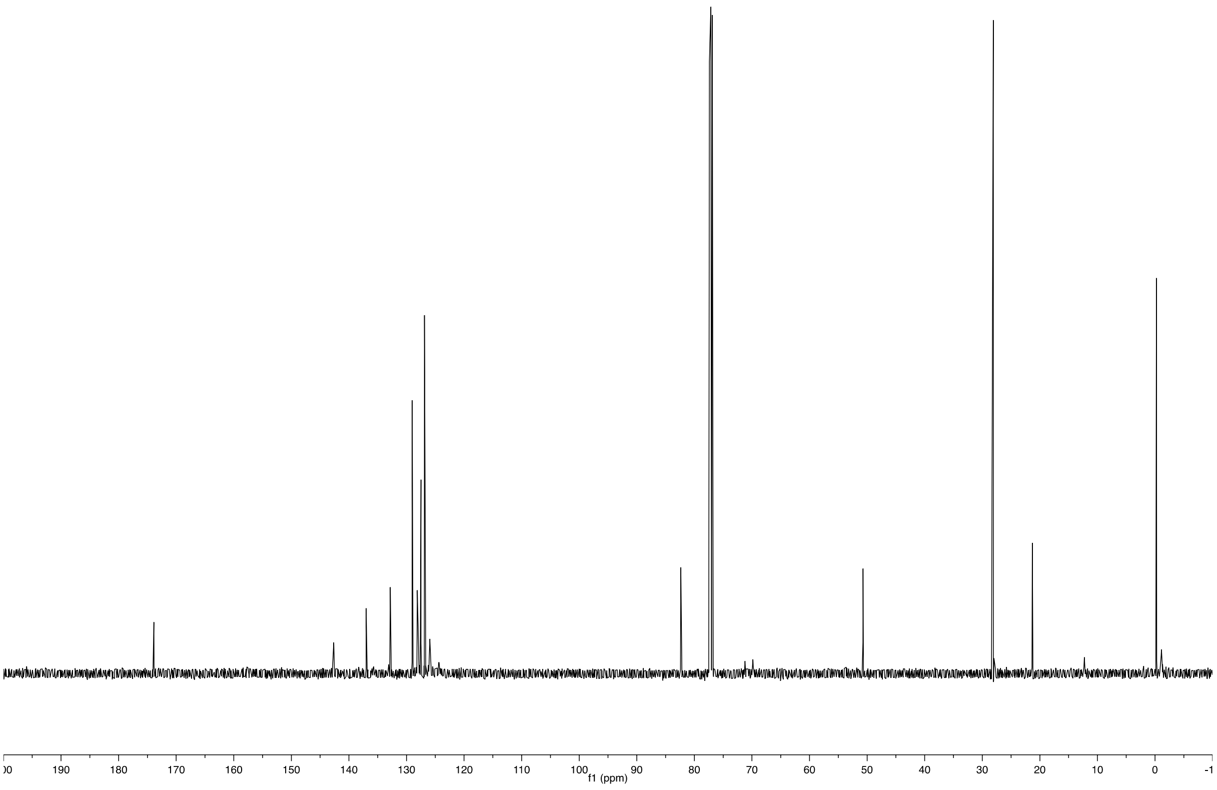

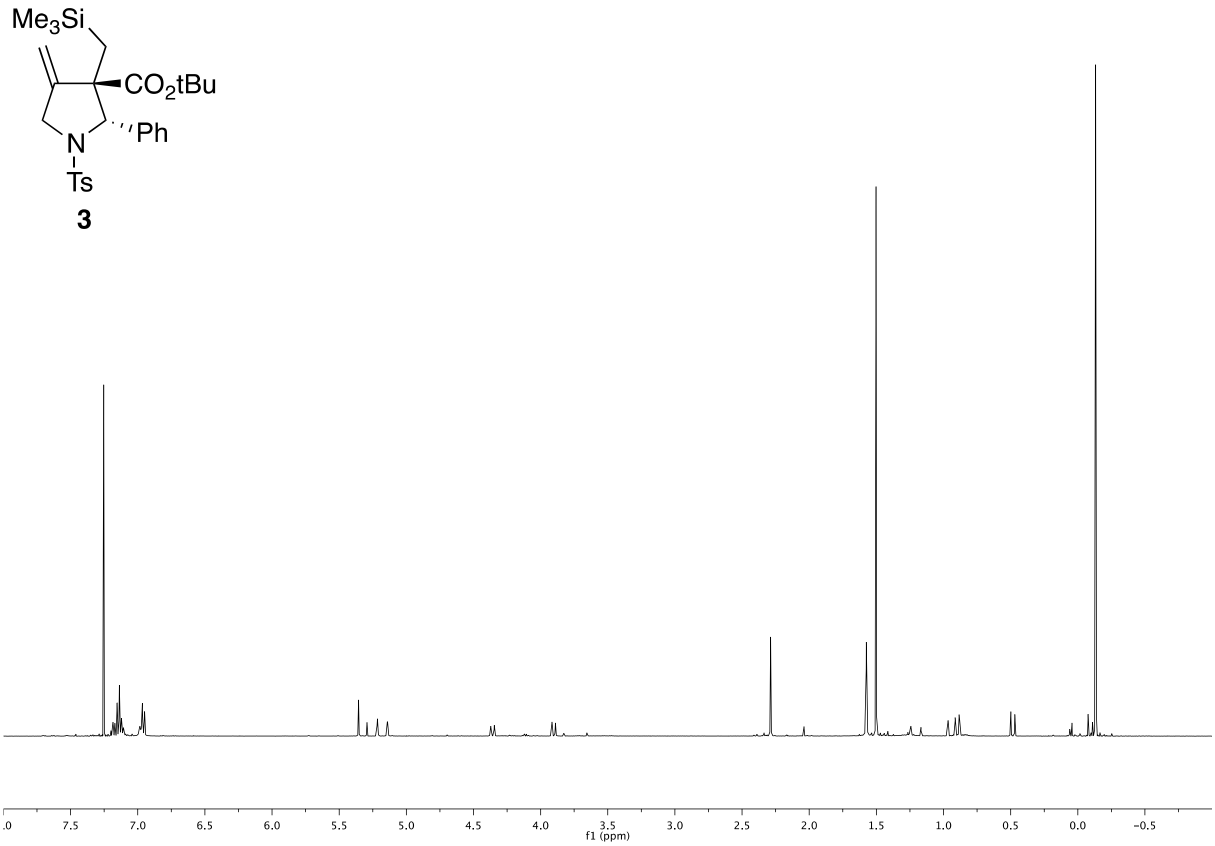

3

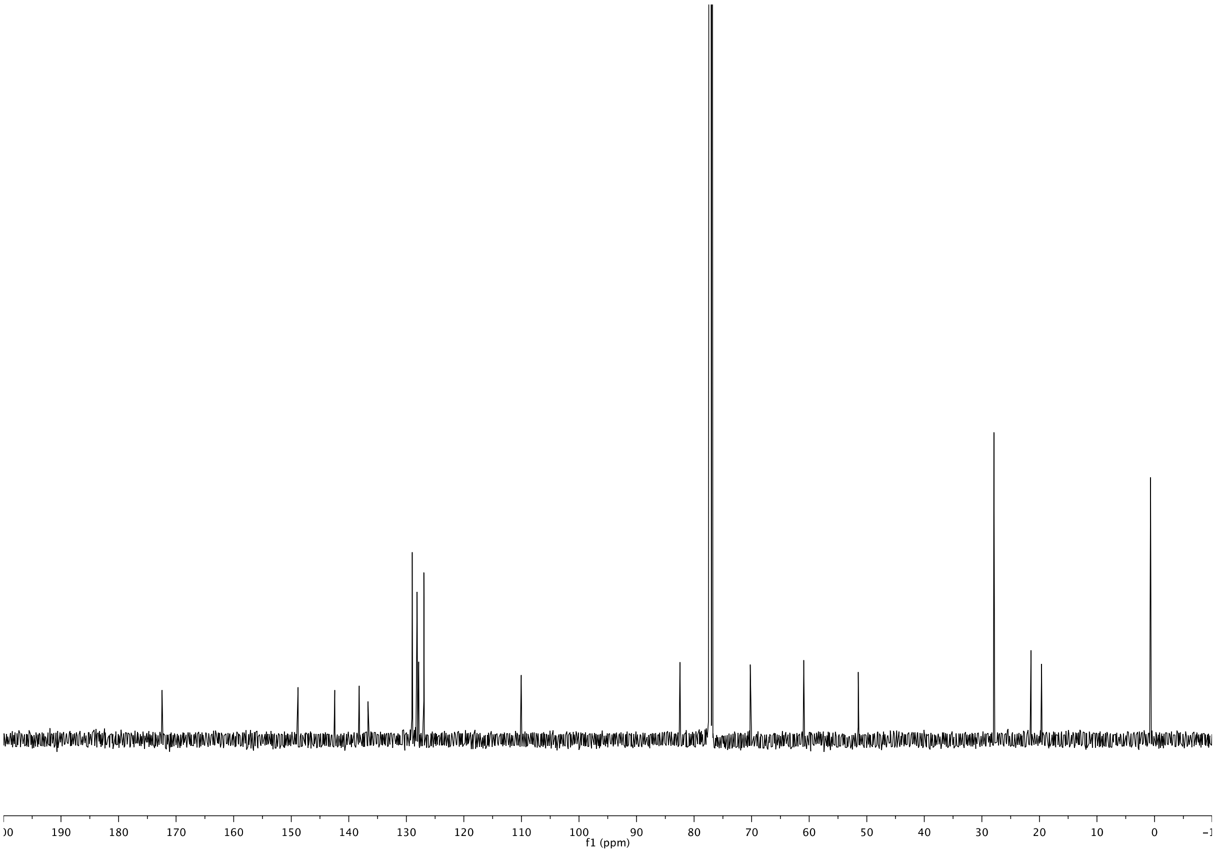

Supplement: File 1 — Experimental procedures and 1H and 13C NMR spectra. [file Beilstein_J_Org_Chem-09-1326-s001.pdf]
